# Structural Mechanism behind Distinct Efficiency of Oct4/Sox2 Proteins in Differentially Spaced DNA Complexes

**DOI:** 10.1371/journal.pone.0147240

**Published:** 2016-01-20

**Authors:** Dhanusha Yesudhas, Muhammad Ayaz Anwar, Suresh Panneerselvam, Prasannavenkatesh Durai, Masaud Shah, Sangdun Choi

**Affiliations:** Department of Molecular Science and Technology, Ajou University, Suwon, 443–749, Korea; University of Minnesota, UNITED STATES

## Abstract

The octamer-binding transcription factor 4 (Oct4) and sex-determining region Y (SRY)-box 2 (Sox2) proteins induce various transcriptional regulators to maintain cellular pluripotency. Most Oct4/Sox2 complexes have either 0 base pairs (Oct4/Sox2^0bp^) or 3 base pairs (Oct4/Sox2^3bp^) separation between their DNA-binding sites. Results from previous biochemical studies have shown that the complexes separated by 0 base pairs are associated with a higher pluripotency rate than those separated by 3 base pairs. Here, we performed molecular dynamics (MD) simulations and calculations to determine the binding free energy and per-residue free energy for the Oct4/Sox2^0bp^ and Oct4/Sox2^3bp^ complexes to identify structural differences that contribute to differences in induction rate. Our MD simulation results showed substantial differences in Oct4/Sox2 domain movements, as well as secondary-structure changes in the Oct4 linker region, suggesting a potential reason underlying the distinct efficiencies of these complexes during reprogramming. Moreover, we identified key residues and hydrogen bonds that potentially facilitate protein-protein and protein-DNA interactions, in agreement with previous experimental findings. Consequently, our results confess that differential spacing of the Oct4/Sox2 DNA binding sites can determine the magnitude of transcription of the targeted genes during reprogramming.

## Introduction

Reprogramming of human somatic cells into induced pluripotent stem cells (iPSCs) will assist in the development of several disease-specific treatments [1]. The generation of iPSCs from somatic cells involves 4 transcription factors, namely, octamer-binding transcription factor 4 (Oct4), sex determining region Y (SRY)-box 2 (Sox2), Kruppel-like factor 4 (Klf4), and the cellular myelocytomatosis oncogene (c-Myc). Among these factors, Oct4 and Sox2 are the major contributors for stem cell reprogramming [1]. Oct4 is a developmental regulator capable of coordinating an array of developmental processes, ranging from the establishment of the embryonic pluripotent ground state to terminal differentiation [2]. Sox2 is crucial for maintaining the pluripotency of undifferentiated embryonic stem cells, such as neural stem cells [3]. Binding of Oct4 and Sox2 to DNA occurs in a sequence-specific manner [3], with Oct4 binding to ATGC(A/T)AAT DNA sequences and Sox2 binding to C(T/A)TTGTT sequences [4].

“Oct4” refers to isoform OCT4A encoded by the POU domain, class 5 transcription factor 1 (*POU5F1*) gene, which is positioned on chromosome 6 in the human genome [5]. Oct4 consists of a POU-specific domain (POU_S_) with 4 α-helices (75 amino acids) and a POU-homeodomain (POU_HD_) with 3 α-helices (60 amino acids) [6]. These 2 domains are connected by a linker region of variable length (approximately 17 amino acids in length) [7]. The linker region of Oct4 forms an α-helical structure in contrast to the unstructured linker region of other POU-family proteins, which makes Oct4 unique in the reprogramming process [6,7]. Experimental data suggests that the linker region mediates the transformation of somatic cells into pluripotent cells [7].

Sox2 was determined from SRY proteins and is a member of the SoxB1 family [3]. SoxB1 family members contain a short N-terminal domain, a high-mobility group (HMG) box domain, and a long C-terminal sequence [8]. The DNA-binding HMG box domain of Sox2 recognizes the minor groove of DNA [3]. The Sox family of transcription factors plays widespread roles in embryonic development; however, Sox2 binding to DNA alone does not initiate transcription, but requires a binding partner at an adjacent site on the targeted DNA [3].

Oct4 can bind to DNA as a monomer, a homodimer, or a heterodimer with other transcription factors such as Sox2 [9]. Although Oct4 and Sox2 play independent roles in determining other cell types, they can promote cell reprogramming through a cooperative interaction between the two proteins, which drives the transcription of target genes [3,8]. The known target genes of Oct4/Sox2 heterodimers include fibroblast growth factor 4 (*Fgf4*), undifferentiated embryonic cell transcription factor 1 (*Utf1*), F-box protein 15 (*Fbxo15*), as well as *Sox2* and *Pou5f1* (the gene encoding Oct4) themselves [9,10]. The interaction interface between the POU_S_ domain of Oct4 and the HMG domain of Sox2 in DNA-bound heterodimers differs depending upon the number of base pairs between their respective DNA-binding sites [11]. The α1 helix of Oct4 and the α3 helix of Sox2 interact with each other after binding to the *Utf1* promoter, where no base pairs separate the Oct4 and Sox2 binding sites [11]. In the case of the *Fgf4* promoter element, 3 base pairs are interspersed between the Oct4 and Sox2 binding sites, which results in an Oct4/Sox2 interaction interface involving the C-terminal loop of Sox2 and the α1 helix of Oct4 [11,12]. Oct4/Sox2 interactions with DNA in biological systems are considered important in reprogramming processes [13].

Molecular dynamics (MD) simulations provide in-depth details regarding the motions of individual atoms of biological macromolecules in an appropriate time scale [14,15]. MD simulations together with binding free energy calculations can provide a quantitative prediction of protein-DNA binding energies [15]. In this study, the structural and functional behavior of Oct4/Sox2^0bp^ (0 base pairs separating the Oct4 and Sox2 DNA-binding sites) and Oct4/Sox2^3bp^ (3 base pairs separating the Oct4 and Sox2 DNA-binding sites) complexes were characterized using MD simulations, principal component analysis (PCA), and dynamic cross-correlation mapping (DCCM). The conformational domain movements of the Oct4 and Sox2 proteins, as well as the secondary structural changes in the Oct4 linker region may cause the complexes to show different activities during the reprogramming process. Curves+ software was used to analyze potential conformational changes occurring in the DNA during Oct4/Sox2 binding. Moreover, binding free energy and per-residue decomposition calculations were performed through molecular mechanics Poisson-Boltzmann surface area (MM/PBSA) determinations and used to characterize the stabilization of protein-DNA interactions.

## Materials and Methods

### Initial structures

The crystal structures of the Oct1/Sox2/Hoxb1 element [12] (PDB ID: 1O4X), Oct1/Sox2/FGF4 [11] (PDB ID: 1GT0), and Oct4/PORE/DNA [7] (PDB ID: 3L1P) were obtained from the Protein Data Bank (PDB). The missing residues (87–89) in the linker region of the Oct4 crystal structure were built into the Oct4/PORE/DNA structure, as described previously [16], and a final model was chosen based on the random forest-based protein model quality assessment (RFMQA) score [17]. The homologous Oct1 and Oct4 transcription factors possess similar DNA-binding specificities [18]. Hence, we modeled these two complexes, (Oct4/Sox2^0bp^ and Oct4/Sox2^3bp^) by superimposing Oct4 with Oct1 in 1O4X and 1GT0 complexes, followed by the removal of Oct1 proteins. The modeled Oct4/Sox2^0bp^ and Oct4/Sox2^3bp^ complexes were further subjected to energy minimization by steepest descent and conjugate gradient, using chimera in order to remove steric clashes generated during structure modeling. Structural inconsistencies were removed by adding hydrogen atoms and partial charges using the Dock Prep application with AMBER force field parameters. The steepest decent with 5000 steps was determined for highly unfavorable clashes, followed by conjugate-gradient calculations with 10,000 steps to reach an energy minima by removing the clashes [19].

For simplicity, we considered two proteins (Oct4 and Sox2) as a single molecule during the simulations. The residues were numbered from 1 to 152 for Oct4 and from 153 to 232 for Sox2. DNA was considered as a separate molecule, and the base pairs were numbered from 1 to 19 for the Oct4/Sox2^0bp^ complex, and from 1 to 22 for the Oct4/Sox2^3bp^ complex.

### MD simulations

MD simulations for both complexes (Oct4/Sox2^0bp^ and Oct4/Sox2^3bp^) were performed with GROMACS 4.6 [20] at neutral pH, using an improved Amber-ff99SB-ILDN force field [21]. The improved force field introduced corrections for DNA, the protein backbone, and the amino acid side chains [22]. The system was solvated with TIP3P water model in a truncated octahedron box using a distance of 1 nm between the complex and the edge of the box. The dimensions of the boxes were 10 × 9 × 8 nm for the Oct4/Sox2^0bp^ complex and 11 × 10 × 9 nm for the Oct4/Sox2^3bp^ complex. The Na^+^ and Cl^-^ ion concentrations were maintained at 150 mM. The LINCS algorithm [23] was used to constrain all bonds, including those of hydrogen atoms. The Particle Mesh Ewald (PME) [24] method was used to evaluate electrostatic interactions. Except for van der Waals interactions, all cutoffs were set to 0.9 nm throughout the simulation. The system was equilibrated under constant temperature (300 K) using V-rescale [20], and pressure (1 bar) for 100 ps, using the Parrinello–Rahman method [20]. MD simulations were performed for 250 ns for each complex under NPT ensemble. The atomic coordinates were saved every 2.0 ps and, thus, 125,000 structures were collected with each system for further analysis.

### Principal component analysis (PCA)

A vivid picture of the complete and correlated motions of atoms in the protein-DNA complex was obtained by PCA. This method was based on constructing a covariance matrix of complex sets of variables [25–27] and was used to reduce the higher-dimension data to extract meaningful information from the protein-DNA complex throughout the simulation.

The ensemble formula used to obtain a covariance matrix with elements C_ij_ for coordinates i and j is given as:

$$C_{ij}=\langle (x_{i}-\langle x_{i}\rangle )\left(x_{j}-\langle x_{j}\rangle \right)\rangle$$ (1)

where, x_i_ and x_j_ are the mass-weighted Cartesian coordinates of the atoms present in the system. Angular brackets “〈 〉” represent the time average. The eigenvectors represent the direction of the coordinated motion of atoms, and the eigenvalues represent the magnitude of the motion along the movement direction [26]. The dynamic motion of atoms in a complex was calculated from their trajectories to define the structural changes of the complex observed during the simulation.

### Dynamic cross-correlation map (DCCM)

A DCCM was generated and used to calculate the time-correlated atomic motion of the system [28,29]. We selected the last 900 snapshots (involving only Cα atoms) at 2.0 ps intervals and subjected them to DCCM analysis. This analysis revealed the largest motions within the system. The DCCM map was calculated as follows [29]:

$$C_{ij}=\frac{\langle \Delta r_{i}.\Delta r_{j}\rangle }{\left(\sqrt{\langle \Delta r_{i}^{2}\rangle }.\sqrt{\langle \Delta r_{j}^{2}\rangle }\right)}$$ (2)

where, Δ*r*_*i*_ and Δ*r*_*j*_ are the displacements from the mean position of *i*-th and *j*-th atom, respectively. The angular brackets “〈 〉” represent the time average over the entire trajectory. *C*_*ij*_ returned either positive or negative values (real numbers). Positive values represented a correlated motion between residues *i* and *j*, and negative values represented anti-correlated motions between residues *i* and *j* [28,29]. The DCCM map was constructed using the ‘bio3d’ module of the R base analysis tool [30].

### Binding free energy calculations

The MM/PBSA method was used to calculate the free energies of molecules in solution including those related to protein-protein, protein-ligand, and protein-DNA binding, which enables analysis of the stabilities of different forms of DNA and RNA molecules [31]. We used open source AMBERTools package (MMPBSA.py) [32] to perform binding free energy calculations and per-residue free energy decompositions. The per-residue free energy decompositions were used to evaluate the contribution of each residue to the total binding free energy of the complex [33]. The energy constituents included molecular mechanics (MM), electrostatic contributions to solvation (PB), and nonpolar contributions to solvation (SA). The binding free energy for protein-DNA complexes was calculated based on the following equation [15,32]:

$$\Delta G_{binding}=\Delta G_{complex}-\Delta G_{protein}-\Delta G_{DNA}$$ (3)

where, ∆*G*_*complex*_ represents the free energy of a DNA-protein complex, and ∆*G*_*protein*_ and ∆*G*_*DNA*_ are the free energies of protein and DNA, respectively.

The vacuum-potential energy, *E*_*Molecular–Mechanics*_, includes the energies of both bonded and non-bonded interactions and is calculated based on the molecular mechanics force field parameters [34], as shown below:

$$E_{Molecular-Mechanics}=E_{bonded}+E_{non-bonded}$$ (4)

where, *E*_*Molecular-Mechanics*_ represents the gas-phase energy, *E*_*bonded*_ represents bonding interactions consisting of the bond, angle, dihedral, and improper interactions. Non-bonded interactions, *E*_*non-bonded*_, include both electrostatic and van der Waals interactions.

In the MM/PBSA approach, the solvation free energy was calculated using an implicit-solvent model. The solvation free energy is given by the following equation:

$$\Delta G_{solvation}=\Delta G_{Polar}+\Delta G_{Non-polar}$$ (5)

where, *∆G*_*Polar*_ and *∆G*_*Non-polar*_ are the electrostatic and non-electrostatic contributions to the solvation free energy, respectively. The electrostatic term, *∆G*_*Polar*_, is estimated by solving the Poisson–Boltzmann (PB) equation. The non-polar solvation energy, ∆G_*Non-Polar*_ is separated into attractive (dispersion) and repulsive (cavity) interactions.

### DNA conformational analysis

Curves+ is a widely used nucleic acid conformation-analysis program, which is applicable to canonical or modified bases and backbone structures [35]. The program enables a comprehensive analysis of DNA structures, base pair parameters, and backbone and groove parameters [35]. A simple matrix-based scheme was used to calculate the complete set of parameters to characterize the orientation and displacement of the base pairs and base pair steps of DNA structures. In addition, this program was used to analyze the MD trajectories.

### Prediction of Oct4/Sox2 binding sites on target genes

The differential spacings between Oct4 and Sox2 DNA-binding sites have been predicted for some target genes [9,36,37]. Possible binding-sequence preferences for the Oct4 and Sox2 proteins were retrieved from the JASPAR database [38]. Preferential binding sequences for Oct4 and Sox2 was identified as [AT]TG[CT][AT][AC][AT][TA] and [CT][TC]TT[GC]T[TC], respectively. The sequences in the square brackets represent possible bases at the preferred binding sites. The sequence manipulation suite (SMS) [39] was used to predict the preferential binding sites of Oct4 and Sox2 and to determine the differential spacings between them. The gene-promoter sequence and preferential binding patterns were given as input to predict the Oct4 and Sox2-binding sites in target genes, as described previously [39].

The UCSF Chimera [19] and Visual Molecular Dynamics [40] packages were used for visual assessment of the trajectory files and to generate images. The DNA-parameter graphs were generated by Matplotlib, and MD simulations were performed using a Dell PowerEdge server with a CentOS6 GNU/Linux operating system.

## Results

### Evaluation of the stability of the Oct4/Sox2^0bp^ and Oct4/Sox2^3bp^ complexes

The Oct4/Sox2^0bp^ and Oct4/Sox2^3bp^ complexes were generated by superimposition of Oct4 with Oct1 in the 1O4X and 1GT0 crystal structures, respectively. The dynamic behavior and structural adaptation of Oct4/Sox2^0bp^ and Oct4/Sox2^3bp^ were analyzed by MD simulation. The final snapshot obtained at the end of the simulations was considered to show a representative structure of all models that were further subjected to energy minimization and consequently used for analysis.

**Fig 1:**
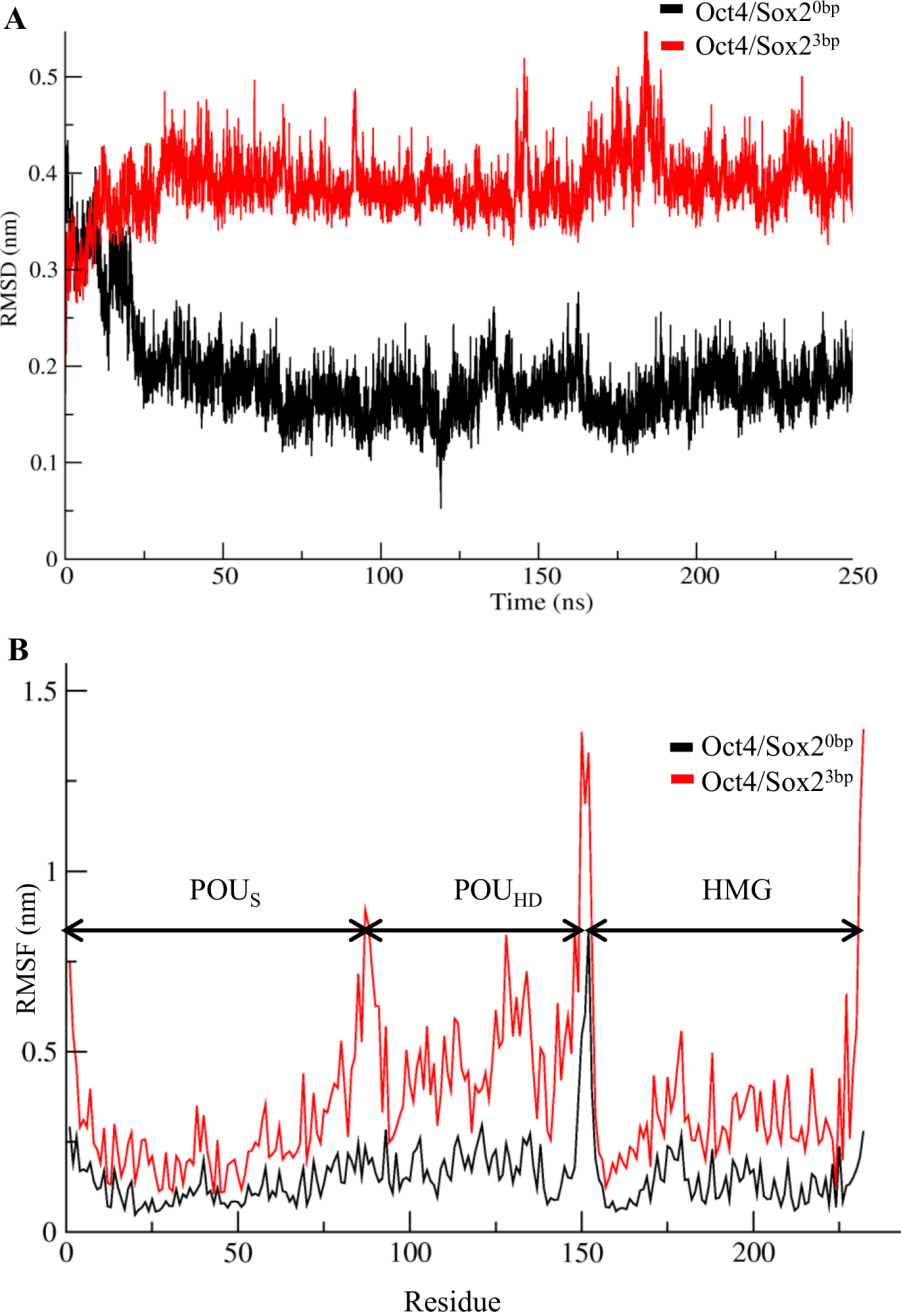
Stability of Oct4/Sox2^0bp^ and Oct4/Sox2^3bp^ complexes. (A) Root mean square deviations (RMSDs) were performed for backbone atoms, with respect to the initial structure, to attain their equilibrium positions and maintain the relaxed state throughout the simulation. (B) Root mean square fluctuation (RMSFs) of protein-backbone residues showing higher fluctuations in the HMG and POU_HD_ domains of Oct4/Sox2^3bp^ compared with those of the Oct4/Sox2^0bp^ complex. Black represents stability of Oct4/Sox2^0bp^ and red represents stability of Oct4/Sox2^3bp^ complex.

The stability of the complexes was measured by root mean square deviations (RMSDs) of the backbone atoms relative to the initial structure. The RMSD of the Oct4/Sox2^0bp^ complex indicated that the complex fluctuated from 0.4 to 0.2 nm until it reached an equilibrium state at 50 ns. The complex was stable thereafter, and the stability was maintained throughout the simulations (Fig 1A). For the Oct4/Sox2^3bp^ complex, the RMSD fluctuated from 0.3 to 0.4 nm up to 60 ns, after which it reached an equilibrium state. However, both complexes showed minor fluctuations at 150 ns. The RMSD graph confirmed that the stability of both complexes was well maintained at a constant temperature and pressure during the simulation (Fig 1A).

Root mean square fluctuations (RMSFs) of the residues revealed that the POU_HD_ and HMG domains of the Oct4/Sox2^3bp^ complex fluctuated more than those in the Oct4/Sox2^0bp^ complex (Fig 1B). The high flexibility of the Oct4/Sox2^3bp^ complex was mainly due to the loosely packed protein arrangement, as well as weak interactions between the Oct4 and Sox2 proteins. In both complexes, the POU_S_ regions did not show much fluctuation, which was mainly due to the strong interactions with DNA [41].

### Structural and dynamic analysis of the Oct4 and Sox2 proteins in the Oct4/Sox2^0bp^ and Oct4/Sox2^3bp^ complexes

**Fig 2:**
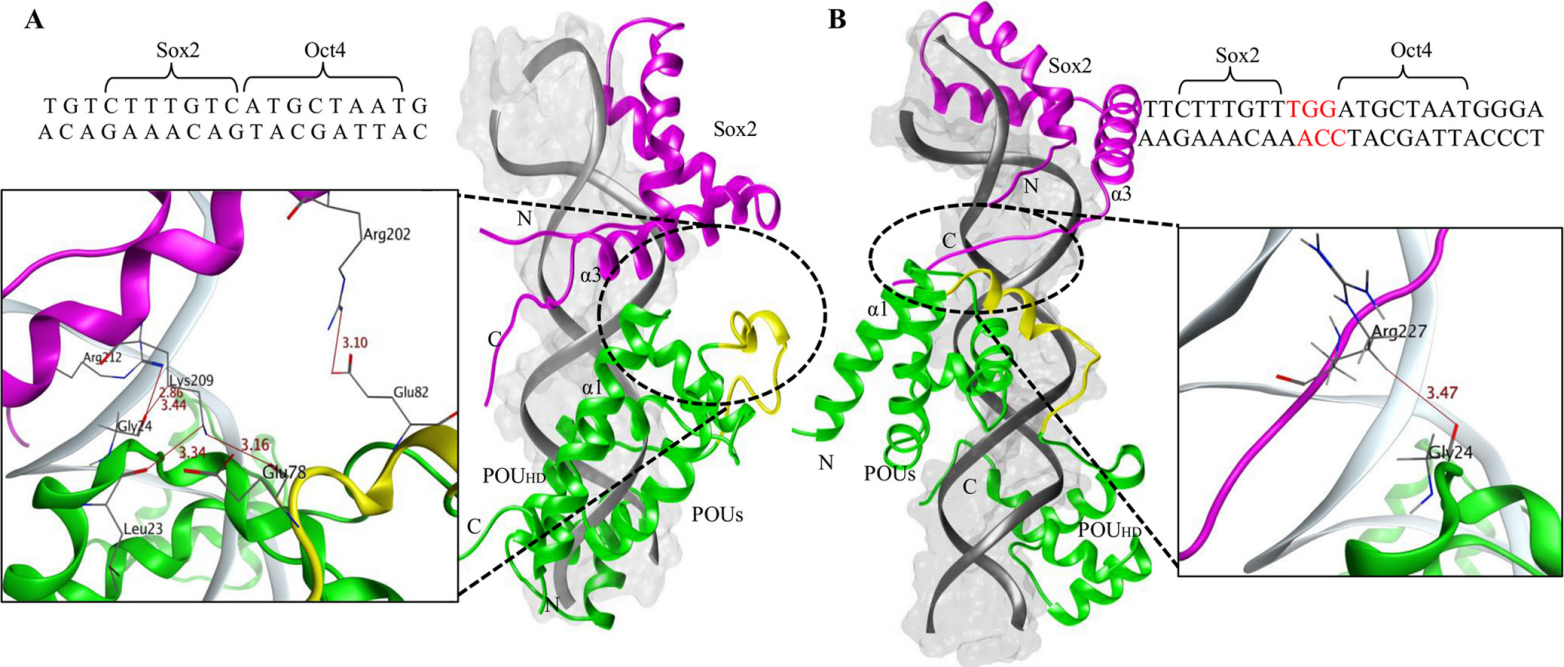
Structural arrangements of the Oct4 and Sox2 proteins. (A) The Oct4/Sox2^0bp^ complex with 0 base pairs separating their DNA-binding sites, in which the POU_S_ α1 helix and the HMG α3 helix are involved in protein-protein interactions. (B) Oct4/Sox2^3bp^ complex with 3 base pairs separating their DNA-binding sites, in which the POU_S_ α1 helix and the C-terminal loop of the HMG domain are involved in protein-protein interactions. Oct4 is depicted in green, Sox2 in magenta, the DNA surface in grey, and the Oct4 linker region in yellow. Hydrogen bond-interacting residues are also indicated for the complexes.

In the case of the modeled Oct4/Sox2^0bp^ complex, the Oct4 and Sox2 DNA binding sites were adjacent. As a result, the binding of Oct4 with Sox2 brings the α1 helix of the POU_S_ domain and the α3 helix of HMG domain close juxtaposition, enabling the formation of a strong, well-packed interface with the DNA (bonding and non-bonding interactions) [12]. Most notably, Gly24 of the POU_S_ domain in the α1 helix established a two-hydrogen bond with the side chains of Lys209 and Arg212 of the HMG domain in the α3 helix. Also, Lys209 of HMG domain established a two-hydrogen bond with Leu23 and Glu78 of POU_S_ domain (Fig 2A). These hydrogen bonds were maintained throughout the simulations (S1A Fig). In addition, Glu82 of the POUs domain in the α1 helix established a hydrogen bond with Arg202 of the HMG domain in the α3 helix, but this interaction was unstable due to conformational changes occurring during the MD simulation. Apart from hydrogen bonding, the complex was also optimized by non-bonding interactions particularly by the residues Ile21, Gly24, Glu51, and Asp75 of Oct4 and Arg206, Glu207, Lys209, Arg212, and Met216 of Sox2. Table 1 shows the residues that contributed to the intra-protein salt-bridge formation, bonding and non-bonding interactions observed during MD simulations in the Oct4/Sox2^0bp^ and Oct4/Sox2^3bp^ complexes.

**Table 1:** Residues of the Oct4/Sox2^0bp^ and Oct4/Sox2^3bp^ complexes involved in bonding and non-bonding interactions.

Complex	Hydrogen bond-interacting residues	Intra-salt bridge-forming residues	Non-bonded interacting residues
**Oct4/Sox2**^**0bp**^	(Gly24) O…N (Lys209)	Lys14-Asp221	Glu8, Lys17*, Gln18, Arg20*,
	(Gly24) O…N (Arg212)	Asp29-Arg202	Ile21, Gly24, Glu51,
	(Glu82) O…N (Arg202)	Glu78-Arg202	Arg64, Asp75, Glu101,
	(Leu23) O…N(Lys209)	Glu78-Arg212	Arg103, Glu109, Asp135,
	(Glu78) O…N(Lys209)	Lys85- Glu198	Arg138, Arg144, Arg206,
		Glu82-Lys209	Glu207, Lys209, Arg210,
		Glu82-Arg202	Arg212*, Ala213, Met216
**Oct4/Sox2**^**3bp**^	(Gly24) O… N (Arg227)	Asp29-Arg225	Lys17*, Arg20*, Asp29,
		Glu82-Arg225	Glu51, Glu101, Arg103,
		Glu87-Lys223	Glu207, Arg210, Lys223*,
			Arg225*, Arg227.

* Protein residues predicted to form contacts with DNA backbone.

The Oct4/Sox2^3bp^ complex differs from the Oct4/Sox2^0bp^ complex by a 3 base pairs insertion between the DNA-binding sites of Oct4 and Sox2 (Fig 2B). The increased gap between the binding sites for these proteins leads to an alternative inter-protein binding surface, as well as changes in the DNA-interaction surface. Thus, the corresponding orientation of Sox2 with respect to Oct4 changes by a 108.2° rotation around the axis of the DNA, which equates to an approximately 36.1° rotation per base pair insertion, based on the known structure of B-DNA [12]. The relative orientation resulting from the 3 base pairs insertion leads to an alignment of Oct4 in an opposite direction relative to Sox2, as depicted in Fig 2B. The 3 base pairs insertion between the binding sites increases the distance between proteins; hence, the chance of strong interactions between Oct4 and Sox2 are lower when compared to the Oct4/Sox2^0bp^ complex.

Interactions between Oct4 and Sox2 were maintained by the partial surfaces of the α1 POU_S_ domain and the C-terminal loop of the HMG domain. Only one hydrogen bond was observed between Gly24 of the POU_S_ domain and Arg227 (side chain) of the C-terminal loop of the HMG domain (Fig 2B). The minimum distance between the residues was maintained throughout the simulations (S1B Fig). Other important residues involved in the non-bonding interactions were Lys17, Arg20, Asp29, and Glu51 of Oct4 and Glu207, Arg210, Lys223 and Arg225 of Sox2. The salt bridge was observed between Arg225 of Sox2 and Asp29, Glu82 of Oct4, and between Glu87 of Oct4 and Lys223 of Sox2 (Table 1).

### Conformational transitions and dynamic domain motions of the Oct4/Sox2^0bp^ and Oct4/Sox2^3bp^ complexes

**Fig 3:**
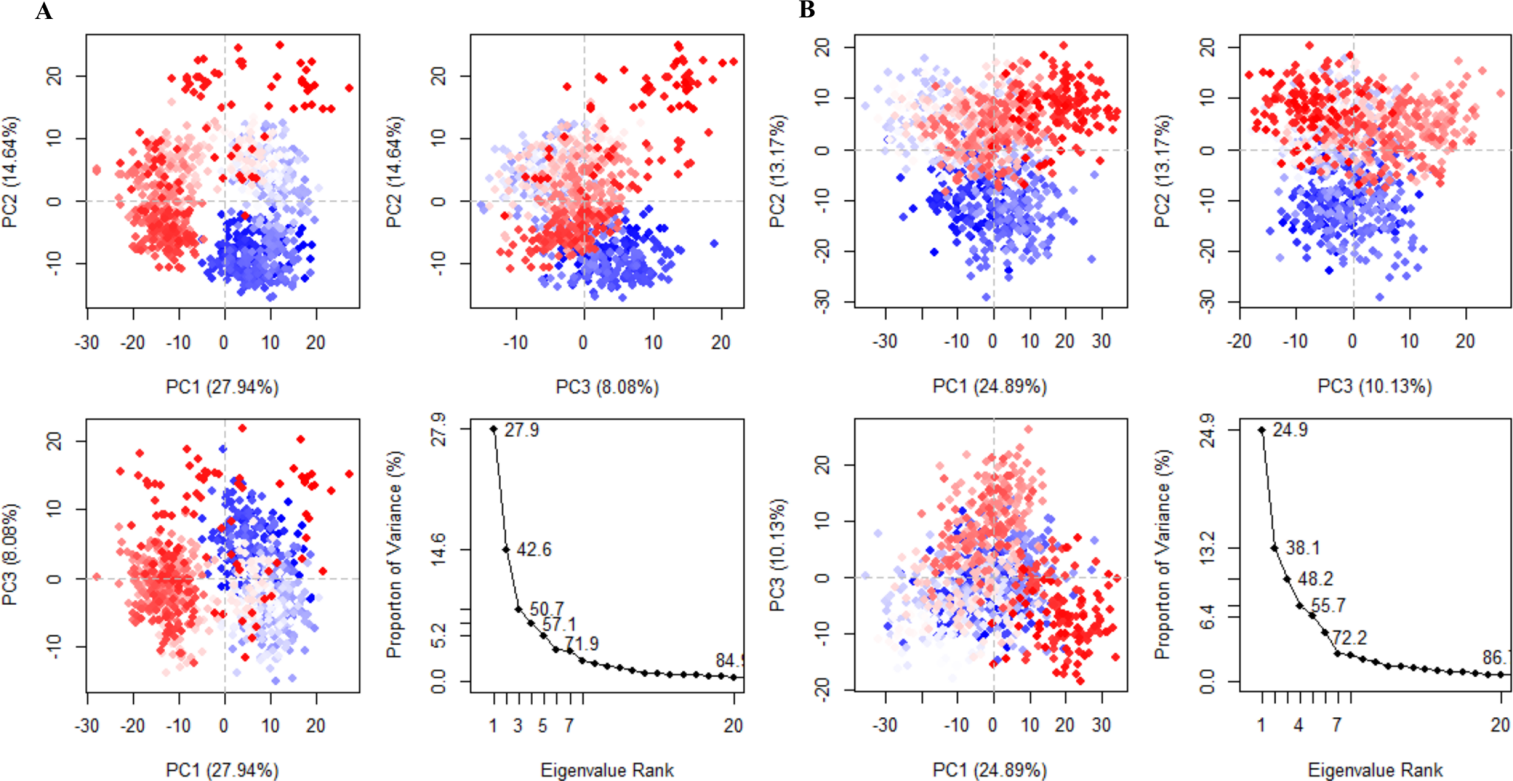
Projections of trajectories onto the subspace by the first three eigenvectors. (A) Projection of trajectories into PC1, PC2, and PC3 for the Oct4/Sox2^0bp^ complex. The converged stable conformation and unstable scattered state are shown with red and blue dots, respectively. (B) Projection of trajectories into PC1, PC2, and PC3 for the Oct4/Sox2^3bp^ complex. Neither conformational state was stable (scattered blue and red regions). The white dots indicate the intermediate states observed in both complexes.

PCA was used to study and analyze the distinct protein conformational states in a principal component (PC) phase space during the MD simulations [25,42]. The conformational transitions of the complexes were studied by projecting their trajectories onto a two-dimensional subspace spanned by the first three eigenvectors (PC1, PC2, and PC3). Fig 3 shows that both complexes attained two conformational states on the subspace (shown in red and blue). The intermediate state located between these two conformations is shown with white dots. The conjoined distributions of PC1/PC2, PC1/PC3, and PC2/PC3 of the Oct4/Sox2^0bp^ complex revealed that the energetically unstable conformational state (scattered blue region) neared convergence and attaining a stable conformational state (compact red region; Fig 3A). Consequently, the complex required a periodic jump between these conformations during protein-protein and protein-DNA interactions (Fig 3A). In case of the Oct4/Sox2^3bp^ complex, the conjoined distributions of the PCs indicated that the conformations were scattered and energetically less stable than the Oct4/Sox2^0bp^ complex (Fig 3B).

**Fig 4:**
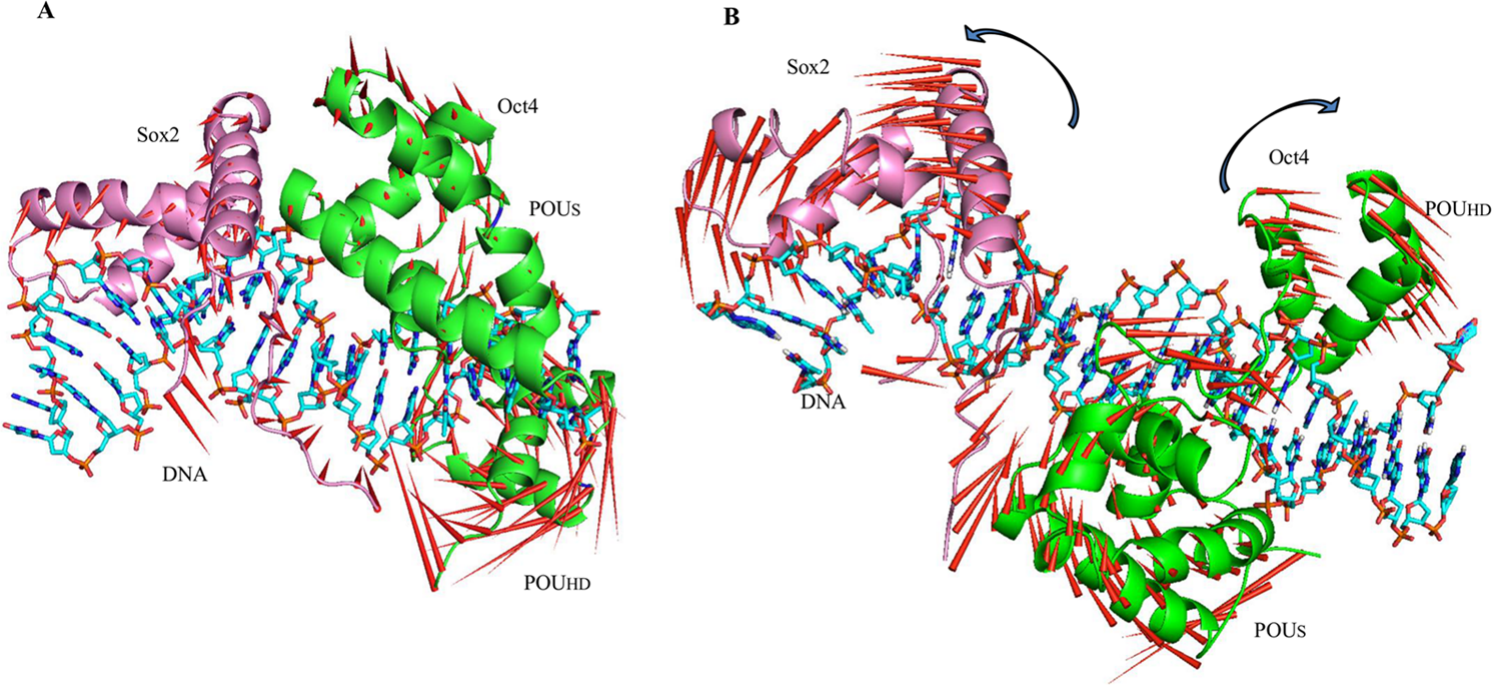
Principal modes of motion for the Oct4 and Sox2 proteins. (A) Dominant motions of Oct4 and Sox2 in the Oct4/Sox2^0bp^ complex. (B) Dominant motions of Oct4 and Sox2 in the Oct4/Sox2^3bp^ complex. The magnitudes and directions of motion of the residues are indicated by red arrows in the ribbon structure. Oct4 and Sox2 are depicted in green and pink, respectively. The DNA is represented as a stick with heteroatom coloring.

The first eigenvector, PC1, reflected large-amplitude motions of the protein backbone conformations, as illustrated in the ‘porcupine plots’ for each complex (Fig 4). For the Oct4/Sox2^0bp^ complex, it was observed that the Oct4 and Sox2 proteins motion was limited due to its strong protein-protein and protein-DNA interactions. Sox2 had a more stable conformation because of its closely packed arrangement [11] and showed a lower magnitude of motion than the Oct4 (Fig 4A). Since the interaction-interface distance between the Oct4 and Sox2 proteins was higher in the Oct4/Sox2^3bp^ complex, the proteins tended to move away from each other (Fig 4B). By measuring RMSFs, it was observed that these regions fluctuate more in the Oct4/Sox2^3bp^ complex than in the Oct4/Sox2^0bp^ complex, which might point to the recruitment of other proteins for stable complex formation [7]. The loosely arranged complex did not appear to restrict the magnitude of motion of Sox2. The direction of motion of the Oct4 linker region in the Oct4/Sox2^0bp^ complex was towards the interacting DNA molecule, but it moved away from the DNA molecule in the case of the Oct4/Sox2^3bp^ complex.

**Fig 5:**
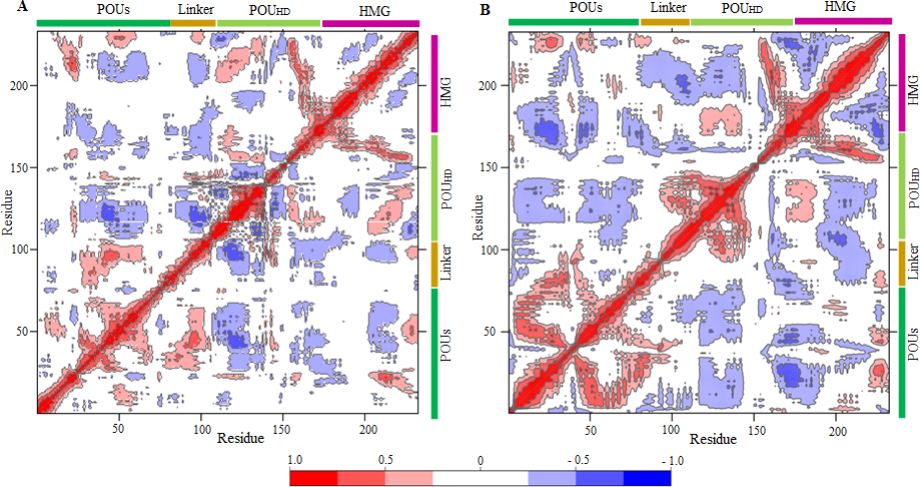
Dynamic cross-correlation map (DCCM). (A) DCCM map for the Oct4/Sox2^0bp^ complex showing positive and negative correlative motions between the Oct4 and Sox2 domains. (B) DCCM map for Oct4/Sox2^3bp^ complex showing positive and negative correlative motions between Oct4 and Sox2 domains. Red represents positive correlations, whereas blue represents negative correlations. The domain regions are labeled.

Correlations between the dynamic motions of the intra- and inter-domains of proteins were quantified through DCCMs [28,29]. In the Oct4/Sox2^0bp^ complex, the Sox2 HMG domain showed a mixture of positive and negative correlative motions within the Oct4 domains (POU_S_ and POU_HD_) and the Oct4 linker region. The Oct4 domains, POU_S_ and POU_HD_, also demonstrated negative correlative motions with respect to each other (Fig 5A). The domain movement of the Oct4/Sox2^0bp^ complex was restricted because of its stable conformation, as depicted in Fig 4A. In the Oct4/Sox2^3bp^ complex, the Sox2 HMG domain showed a negative correlative motion with POU_HD_ and POU_S_ domains. In addition, Sox2 displayed a partially negative correlative motion within the linker region (Fig 5B). The domain movements of the Oct4/Sox2^3bp^ complex were negatively correlated with each other, and opposite directions of motion were observed (Fig 4B).

In addition to the above analysis, we have also predicted that differential spacing occurs between the Oct4 and Sox2 binding sites for other target genes [9,36,37], using SMS. These predictions may provide a better understanding of the nature of Oct4/Sox2 interactions at different target genes (S1 Table).

### Deterministic Oct4 linker region facilitates the different behavior of the Oct4/Sox2^0bp^ and Oct4/Sox2^3bp^ complexes

The Oct4 linker region (76–92 amino acids in Oct4) is a flexible segment that wraps around the DNA and functions to bridge the POU_S_ and POU_HD_ domains [7]. The unique N-terminal part of the linker region in Oct4 is folded in an α-helix, which acts as an interaction interface between proteins and plays a vital role during reprogramming by engaging epigenetic members to Oct4 target genes [7].

**Fig 6:**
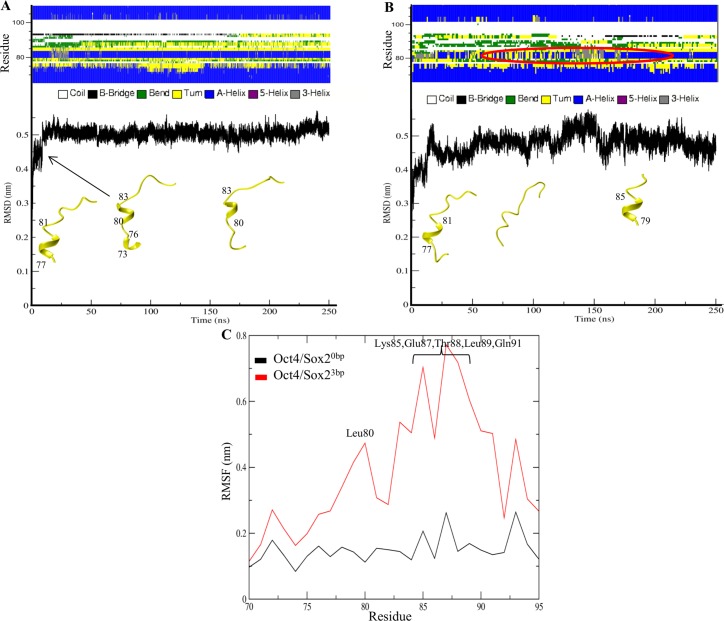
Secondary structure changes and residue fluctuations of the Oct4 linker. (A) Time evaluation of secondary-structure changes, along with RMSD analysis of the Oct4/Sox2^0bp^ linker region. (B) Time evaluation of secondary structure changes, along with RMSD analysis of the Oct4/Sox2^3bp^ linker region. The secondary structure changes observed for the Oct4/Sox2^3bp^ complex are marked in red. (C) RMSFs of linker-region residues 76–92 calculated for 250 ns for both complexes. The residues with relatively high fluctuations for the Oct4/Sox2^3bp^ complex are indicated.

Fig 6A and 6B show the computed secondary structural changes in the linker region of both complexes. The Oct4/Sox2^0bp^ and Oct4/Sox2^3bp^ complexes have an α-helix between residues 77–81 in the initial structure. We examined secondary structure changes during the course of simulation. In the Oct4/Sox2^0bp^ complex, an α-helix was observed between residues 73–76 and 80–83 after a 20-ns simulation. Subsequently, the helix region between 73–76 residues disappeared, but residues 80–83 did not lose their helicity (Fig 6A). For the Oct4/Sox2^3bp^ complex after a 20-ns simulation, residues 80–83 attained a helical conformation and it sustained up to 50 ns. After 50 ns, the complex failed to maintain the α-helix conformation, but after 160 ns, the helical structure was observed again, this time within residues 79–85 (Fig 6B).

Esch *et al*. [7] reported the importance of the linker region in maintaining pluripotency. The length of the Oct4 linker region ranges from 76–92 residues, which is shorter than the unconstrained Oct1 linker region [7]. The reprogramming property of Oct4 can be abolished by point mutations in the linker region [7]. Hence, the analysis of the linker region for the Oct4/Sox2^0bp^ and Oct4/Sox2^3bp^ complexes is of particular interest, and residue fluctuations in the linker regions of both complexes were analyzed in detail. The equilibrium trajectory of linker-region residues in the Oct4/Sox2^3bp^ complex showed fluctuations at residues 80–91 (Fig 6C). The RMSFs of linker-region residues in the Oct4/Sox2^3bp^ complex rose from 0.15 to 0.7 nm, whereas these fluctuations for the Oct4/Sox2^0bp^ complex increased from 0.15 to 0.18 nm. In the case of the Oct4/Sox2^0bp^ complex, residue Glu82 of the Oct4 linker formed a salt-bridge interaction with Arg202 of Sox2 (S2 Fig). In addition, Gln91 formed a hydrogen bond with DNA at position DG25 (S2 Fig). In contrast, none of the interacting partners of the Oct4/Sox2^3bp^ complex in the linker region were observed to form hydrogen-bond interactions. Hence, the fluctuations of linker-region residues for Oct4/Sox2^3bp^ were greater than those of Oct4/Sox2^0bp^, which may translate into an increased stability of protein-DNA complexes. Collectively, the secondary structure changes and fluctuations of the linker region may account for the different stabilities and pluripotency-inducing potentials of each complex.

### Transcriptional mechanism of Oct4/Sox2-induced reprogramming, as predicted by MD simulations

The DNA conformation in both complexes was a perfect B-form double helix with bending in the Sox2-binding site that optimized the protein-DNA interface [12,43]. The formation of hydrogen bonds between amino acid side chains and hydrogen-bond donors and acceptors of individual base pairs confirmed the occurrence of DNA sequence-specific interactions [43,44]. Furthermore, the protein-DNA interaction was stabilized by the penetration of arginine into the minor groove of the DNA-binding site [45].

As observed in our DNA-conformation analysis, a bend occurred in the DT5.DA34 and DT6.DA33 regions of the Oct4/Sox2^0bp^ complex. Some residues interacting with DNA at the Sox2-binding site were Arg167, Arg171, Ser186, Trp193, and Tyr224 and those of the Oct4-binding site were Thr45, Arg49, Ser56, Lys94, Arg95, Arg97, Asn143, and Gln146. A DNA-skeleton structure with these interacting residues is illustrated in the figure (S3A Fig).

Regarding the Oct4/Sox2^3bp^ complex, the bending-interaction interface was located to the DT4.DA43 and DT5.DA42 positions, where the Arg154 and Arg167 residues penetrated into the DNA base pairs, supporting their interaction stabilities. Arg154, Arg157, Asn160, Arg167, Ser183, and Ser186 were some of the major DNA-binding residues of Sox2, and Gln44, Thr45, Arg49, Ser56, Arg95, Arg105, and Gln146 were some of the major Oct4 residues that participated in DNA-binding interactions (S3B Fig). In addition, the residues Arg20, Arg225, Lys223, and Arg228 were involved in hydrogen bonding with bases DG11, DG10, DT9 and DT9, respectively (at the 3 base pairs separated binding sites) and may play an important role in heterodimerization of the Oct4/Sox2^3bp^ complex (S4 Fig).

Hydrogen-bond interactions were studied to explore DNA-protein interactions, as summarized in S2 Table. AT-rich sequences are generally more flexible [45] and, hence, bending occurred at the AT-rich site for both complexes. Experimental evidence indicated that the 3 base pairs separated complex Oct1/Sox2/FGF4 exhibited bending in DC3.DG47 and DT6.DA44, as well as compression in the DT6.DA44 and DG7.DC43 regions [11]. However, no experimental evidence of interacting residues and bending sites has been reported for the Oct4/Sox2^0bp^ complex.

**Fig 7:**
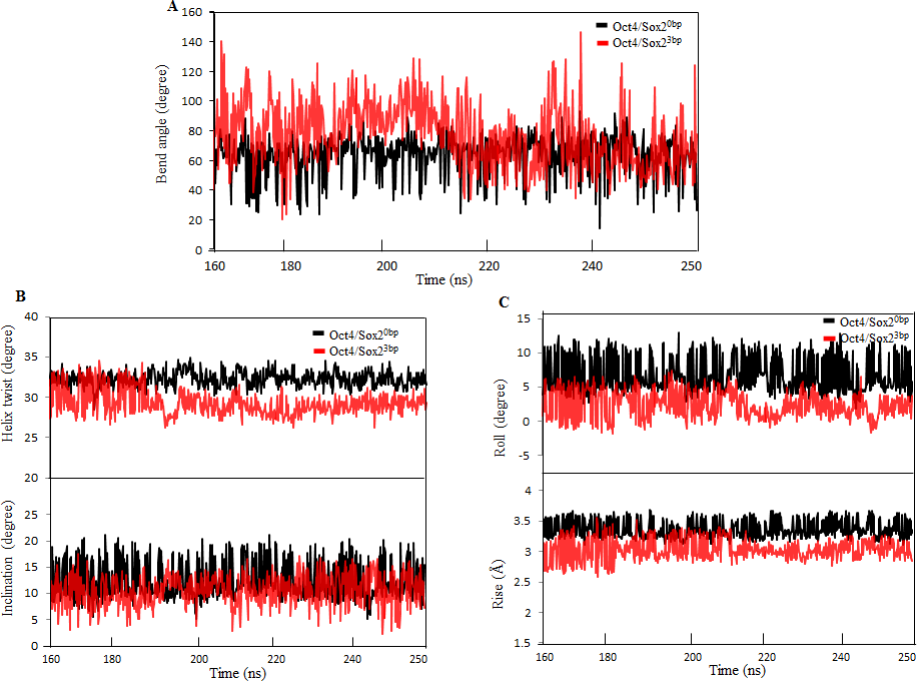
Comparison of DNA parameters. (A) DNA-bend angles for the Oct4/Sox2^0bp^ and Oct4/Sox2^3bp^ complexes. (B) Helix twist and inclination for the Oct4/Sox2^0bp^ and Oct4/Sox2^3bp^ complexes. (C) Rises and rolls for the Oct4/Sox2^0bp^ and Oct4/Sox2^3bp^ complexes. Black represents the Oct4/Sox2^0bp^ complex, and red represents the Oct4/Sox2^3bp^ complex.

Sox2 binding is known to bend DNA to varying extents [3,46]. The average bend angle was calculated between the first and last base pairs of DNA helical segments [47,48]. In MD simulation for the Oct4/Sox2^0bp^ complex, Sox2 binding caused the DNA to bend between 40° and 70° throughout the simulation, and the bending was constant because of the tightly packed arrangement of Sox2 and its strong interactions with the DNA (Fig 7A). The DNA-bend angle for the Oct4/Sox2^3bp^ complex was found to be between 60° and 100° (Fig 7A), with mild fluctuation during the simulations, as observed with the PCA data (Fig 4B). The observed DNA-bend angles for both complexes were in agreement with previous experimental observations [11,12]. Although the complexes had a perfect B-form DNA in their crystal structures [12], both DNAs underwent conformational changes during MD simulations. For the Oct4/Sox2^0bp^ complex, the helix twist and inclination parameter were found to be 33° and 15°, respectively (Fig 7B). In addition, the rise value was 3.4 Å and the roll parameter was 9° (Fig 7C). The Oct4/Sox2^3bp^ complex showed a helix twist ranging from 30° to 33°, an inclination of 10°, a rise of 2.7 Å to 3.1 Å, and a roll of 2° to 5° (Fig 7B and 7C). The perfect A-form DNA had a helix twist of 33°, an inclination as 21°, a rise value as 2.56 Å, and a roll as 6° [49,50]. Thus, the DNAs of these complexes were beginning to make a conformational switch from the B form to the A form during the simulation (Fig 7B and 7C).

### Binding affinity analysis of protein-DNA complexes

With the Oct4/Sox2^0bp^ complex, binding free energy calculations showed that most favorable contributions to the binding process arose from non-bonding electrostatic interactions. The polar solvation energy, which is an unfavorable contribution to the binding free energy, appeared to be highly positive. Nonpolar interactions favorable for the binding process are normally restrained by polar solvation interaction in protein-DNA complexes [15]. The total binding free energy for the Oct4/Sox2^0bp^ complex was calculated as -216.208 kcal mol^−1^, whereas the total binding free energy of the Oct4/Sox2^3bp^ complex was -165.829 kcal mol^−1^. Both complexes showed favorable van der Waals, electrostatic, and non-polar energy values (Table 2). Thus, the binding free energy of Oct4/Sox2^0bp^ was higher than that of the Oct4/Sox2^3bp^ complex.

**Table 2:** Binding free energy of the Oct4/Sox2^0bp^ and Oct4/Sox2^3bp^ complexes calculated using the molecular mechanics Poisson-Boltzmann surface area (MM/PBSA) method.

Energy Component	MMPBSA (kcal mol^-1^)			
	Oct4/Sox2^0bp^		Oct4/Sox2^3bp^	
	Average	Std. Dev.	Average	Std. Dev.
van der Waals energy	-325.2990	7.0953	-361.9749	7.0419
Electrostatic energy	-17342.2697	54.0053	-19514.7821	57.0299
Polar solvation energy	17231.2471	49.2676	19464.7039	47.5960
Nonpolar solvation energy (cavity interaction)	-220.4247	0.7906	-253.4184	2.3159
Dispersion energy	440.5378	1.5269	499.6418	4.6822
*E*_*Molecular–Mechanics*_	-17667.5687	51.7713	-19876.7571	55.8825
∆*G*_*solvation*_	17451.3603	48.8451	19710.9274	46.8390
**∆*G***_***binding***_	**-216.2084**	**10.0798**	**-165.8297**	**16.8724**

### Per-residue free energy contributions in protein-DNA complexes

To characterize and identify the key residues of the Oct4 and Sox2 proteins in both complexes, per-residue free energy decompositions was performed to obtain their individual residue energy contributions, as shown in S3 Table. In the Oct4/Sox2^0bp^ complex, residues Arg20, Arg95, Arg97, Asn143, Arg154, Arg157, Arg171, and Arg227 were among the major contributors for protein-DNA interactions with favorable decomposition free energies. In the Oct4/Sox2^3bp^ complex, residues Arg20, Lys40, Arg95, Arg97, Arg145, Arg154, Lys156, Arg157, Arg227, and Arg228 were potentially important (S3 Table). Other residues such as Arg, Lys, Pro, Gln, Thr, and Asn also participated in protein-DNA interactions for both complexes. The key residues identified by the per-residue decomposition assay, as well as previously reported residues, [7,11,12] are discussed below.

Previous experimental data demonstrated that replacing Lys57 (designated as Lys209 in this study) with Glu57 caused unfavorable charge-charge repulsions [4]. Results from our per-residue decomposition assay showed that residue Lys209 provides structural stability by contributing binding affinities of -0.136 kcal mol^-1^ and -0.252 kcal mol^-1^ to the Oct4/Sox2^0bp^ and Oct4/Sox2^3bp^ complexes, respectively (S3 Table). The crystal structure of the Oct1/Sox2/FGF4 complex showed that salt-bridge formation between these proteins is mainly facilitated by residue Arg75 of Sox2 (Arg227 in our study) and Asp29 of Oct4 [11]. However, residue Asp29 did not noticeably contribute to the binding free energy of both complexes. Residue Arg227 is an important residue for protein-DNA interactions with decomposition energy of approximately -12 kcal mol^-1^ in both complexes.

The key residues Lys17, Arg20, Arg212, Arg227, and Arg228 of both complexes were highlighted in our non-bonding interaction studies (Table 1). The binding-affinity calculations and decomposition energy values highlighted differential behaviors of critical amino acids in the Oct4/Sox2 complexes involved in DNA recognition.

The binding free energy calculations, which were initially performed using the GROMACS tool (g_mmpbsa) [51], gave higher binding free energy values, which may be due to limitations or parameter settings with this tool when calculating protein-DNA affinities. Hence, the results from g_mmpbsa (S4 Table) were considered imprecise, and AMBERTools (MMBPSA.py) was used to calculate binding free energies, due to its advanced algorithm and its wide acceptance by users.

## Discussion

In this study, the structural orientation and cooperative binding of Oct4 and Sox2 in the Oct4/Sox2^0bp^ and Oct4/Sox2^3bp^ complexes were analyzed using MD simulations. The experimental results by Jauch *et al*. [52] suggested that strong cooperative binding occurred between Oct4 and Sox2 in canonical and compressed elements [12,52]. However, the Oct4/Sox2 heterodimerization considerably decreased when 1 or 2 base pairs were added in the DNA-binding site interface [52]. This decrease in heterodimerization may be due to the rotational positioning of the Oct4 and Sox2 proteins with the insertion of intervening base pairs (36.1° per base pair insertion) [12]. The rotational positioning of all these complexes is represented in S5 Fig. Interestingly, the heterodimerization was not affected when 3 base pairs were included in the interface [52]. In addition, experimental data suggest that a lower quantity of Sox2 is required for Oct4-Sox2 heterodimerization in the Oct4/Sox2^0bp^ complex than is required for Oct4/Sox2^3bp^ [52]. Moreover, the induction efficiency was higher for the former than the latter [11,12,52]. Our MD-simulation results and structural-analysis data explain why the Oct4/Sox2^0bp^ complex shows greater induction rate than the Oct4/Sox2^3bp^ complex, suggesting the importance of structural modifications in the process.

Sox proteins shape the DNA-binding interface and facilitate the consecutive recruitment of other transcriptional regulators [3]. Thus, different Sox-family proteins may bend the DNA to various angles and cause diverse biological responses [8]. Moreover, Scaffidi *et al*. [53] reported that a single-nucleotide substitution in the Sox2-binding site caused the DNA molecule to bend differently, resulting in a different transcriptional magnitude [53]. The crystal structure illustrated that Sox2 bends DNA between 50° and 90°, whereas our MD simulation showed that the bend angle was maintained at approximately 45° to 80°, for both complexes (Fig 7A), which is in agreement with previous experimental results [11,12]. In addition, Sox2 binding bends the DNA and thus causes it to unwind at the Sox2-binding site [11]. The helix twists (~33°) and roll parameters (~6°) (Fig 7B) indicated that the DNA in both complexes matched with the characteristics of A-form DNA [49,50], while the rise (~3.1Å) parameter indicated that the DNAs adopted B-form characteristics (Fig 7C). Although the crystal structure for both complexes showed that the associated DNA was in the B-form [12], the DNA underwent a conformational change from B-DNA to A-DNA during our MD simulation.

Proteins are generally dynamic in nature. When they bind to DNA, the protein-DNA interaction alters the protein conformation towards an energetically favorable conformation to support transcription [54]. Because the Oct4/Sox2 complex plays an important role in reprogramming, understanding the local and global transitions of this complex is critical. During our MD simulations, we found that the conformation of Oct4/Sox2^0bp^ complex undergoes a transition state (Fig 3A, from blue to red conformation), including an equilibrium shift required for protein-protein and protein-DNA interaction. After the transition, the complex becomes converged and reaches stable conformational state (Fig 3A). In contrast, the Oct4/Sox2^3bp^ complex transitions through a wider range of conformational states (Fig 3B) and failed to obtain a converged or stable energy state. In addition, the Oct4/Sox2^3bp^ complex underwent increased fluctuations in HMG and POU_HD_ domains (Fig 2B) because of its weaker protein-protein interactions. Therefore, the complex may require additional epigenetic factors to achieve a stable conformational state.

PC1 captures the highest and most meaningful conformational motions, clearly explaining the transitional motions of Oct4 and Sox2 proteins. The Oct4 and Sox2 proteins in the Oct4/Sox2^0bp^ complex were experiencing limited motion mainly because of its stable protein-protein and protein-DNA interactions (Figs 4A and 1B). In contrast, with the Oct4/Sox2^3bp^ complex, both the Oct4 and Sox2 proteins tended to move away from each other with respect to the DNA, plausibly weakening the protein-protein and protein-DNA interactions (Fig 4B). Furthermore, we compared our PCA results with those of the DCCM analysis. The DCCM map of the Oct4/Sox2^0bp^ complex revealed restrictive positive and negative correlative motions between Sox2 HMG domain and the POU_HD_ and linker domains of Oct4 (Fig 5A), owing of its stable conformation upon DNA binding (Fig 4A). In contrast, the Oct4/Sox2^3bp^ complex showed negative correlative motions between the Sox2 and Oct4 domains (Fig 5B), and hence they moved in opposite directions (Fig 4B). Our PCA observations were further supported by the results of the DCCM analysis.

The unique linker region, which determines the reprogramming efficiency of the Oct4 protein, is structured as an α-helix (α5) and functions to attract other epigenetic factors to Oct4 for reprogramming [7]. Thus, the changes observed in the Oct4 linker region during the MD simulations are important. In the crystal structure, the Oct4 linker formed an α-helix between residues 76–82. The Oct4/Sox2^0bp^ complex showed a stable helical structure in the linker throughout the simulation (Fig 6A). In contrast, the Oct4/Sox2^3bp^ complex maintained an α-helix during the initial stage of simulation, lost its helical structure after 50 ns, and regained the helical structure after 160 ns, indicating a higher degree of structural fluctuations in the linker when the interacting proteins had greater separation (Fig 6B). The unconstrained Oct4 linker residues of the Oct4/Sox2^3bp^ complex observed in the RMSF graph provided evidence of its higher flexibility (Fig 6C). MD simulation studies on other proteins have shown that flexible regions play an important role in recruiting other proteins [7,55]. Based on those observations, we hypothesize that linker region may also recruit other epigenetic factors.

Based on the structural dynamics, we observed the non-converged conformational state, opposing Oct4 and Sox2 domain movements, and highly flexible Oct4 linker region that are specific for the Oct4/Sox2^3bp^ complex. Due to this specificity, this complex may recruit other possible epigenetic factors and facilitate the reprogramming process. The whole complex-formation process may take place through sequential events that lead to reduced efficiency during reprogramming. However, for the Oct4/Sox2^0bp^ complex, stable complex formation occurred, which was oriented towards the DNA center, and there may be no need for the complex to recruit other proteins for stabilization. Thus, the induction rate is higher for this complex. The binding free energy calculations indicated that both complexes showed stable protein-DNA interactions with favorable binding free energy values (Table 2). The protein-protein and protein-DNA interacting residues along with the key residues identified from the per-residue decomposition assay are in agreement with previous experimental data [7,11,12].

An increasing number of reports on the structural analysis and MD simulations of transcription factors have provided deeper understandings in the field of computational biology [56–58]. Moreover, results from a recent MD-simulation study demonstrated that the mechanism of Oct4 binding to DNA is modified by Sox2 [59]. In agreement, our MD-simulation study on Oct4/Sox2^0bp^ and Oct4/Sox2^3bp^ complexes showed that structural changes in the Oct4 and Sox2 proteins are responsible for their differences in induction rates during reprogramming. Furthermore, naturally occurring Oct4/Sox2 target genes are observed to have 0-base pairs separations at their binding sites [9,37], which explains the more prevalent and prominent nature of Oct4/Sox2^0bp^ complex. Nevertheless, further analysis of the predicted target genes (S1 Table) with differentially spaced Oct4/Sox2 binding sites may provide a better understanding of the structural and functional behaviors, and efficiencies of the Oct4/Sox2 complex.

Overall, the mechanism behind the distinct efficiencies of differentially spaced Oct/Sox2 complexes at the molecular level was analyzed. It is concluded that the Oct4/Sox2 DNA-binding sites with 3 intervening base pairs results in comparatively less stable conformation states because of the unstructured and flexible Oct4 linker region. This unstable linker may require additional binding partners to attain a stable conformation and to cause a higher induction rate during reprogramming. However, the Oct4/Sox2 binding site with 0 intervening base pairs showed an overall stable conformation including the Oct4 linker region, which potentially enables the complex to promote efficient induction during reprogramming. Computational approaches are helpful in understanding the mechanisms underlying experimental data and structure-function relationships between stem cell factors, which pave the way for advances in stem cell biology.

## Supporting Information

S1 Fig Minimum distance between hydrogen bond-interacting residues. (A) Black represents the minimum distance between Gly24 of Oct4 and Lys209 of Sox2, red represents the minimum distance between Gly24 of Oct4 and Arg212 of Sox2, Green represents the minimum distance between Leu23 of Oct4 and Lys209 of Sox2, blue represent the minimum distance between Glu78 of Oct4 and Lys209 of Sox2 for the Oct4/Sox2^0bp^ complex. (B) The minimum distance between hydrogen bond-interacting residues (Gly24 of Oct4 and Arg227 of Sox2) for the Oct4/Sox2^3bp^ complex is indicated in black.
(TIFF)

S2 Fig Minimum distance between interacting residues in the Oct4 linker region of the Oct4/Sox2^0bp^ complex. The minimum distances for hydrogen bond-interacting residues between Gln91 of the Oct4 linker and the DG25 nucleotide are shown in black. The minimum distances between the salt bridge-forming residues Gln82 (Oct4 linker) and Arg202 (Sox2) are shown in red.
(TIFF)

S3 Fig Oct4 and Sox2 protein interactions with DNA. (A) Oct4 and Sox2 residues interacting with DNA in the Oct4/Sox2^0bp^ complex. (B) Oct4/Sox2 residues interacting with DNA in the Oct4/Sox2^3bp^ complex. Asterisks indicate residues interacting with DNA at the binding site separated by 3 base pairs (red-colored region). DNA is represented as a skeletal structure in grey, and prominently interacting residues are labeled.
(TIFF)

S4 Fig Minimum distances between hydrogen bond-interacting residues and DNA at the binding sites separated by 3 base pairs. The minimum distance between hydrogen bond-interacting residues Lys223 (Sox2), Arg228 (Sox2); Arg225 (Sox2); Arg20 (Oct4); and DT9, DT9, DG10, and DG11 at the 3 base pairs-separated binding site for Oct4/Sox2^3bp^ are shown in blue, black, red, and green, respectively.
(TIFF)

S5 Fig Rotational positioning of base pairs separating the Oct4/Sox2 complexes. (A) Vertical representation of the Oct4 and Sox2 proteins with 0, 1, 2, or 3 base pairs insertions at their binding sites. (B) Horizontal representation of the Oct4 and Sox2 proteins with 0, 1, 2, or 3 base pairs insertions at their binding sites. The insertion of 1 base pair rotates the complex by 36° along the DNA helical axis, facilitating different rotational positioning of POU_S_ with respect to the HMG box domain. The DNA is represented in grey, Sox2 is in magenta, and the Oct4 domains are in green.
(TIFF)

S1 Table Differentially spaced Oct4 and Sox2-binding sites for target genes. (DOCX)

S2 Table Hydrogen bond interactions between DNA and Oct4/Sox2 complexes. (DOCX)

S3 Table Relative binding free energy for residues of the Oct4/Sox2^0bp^ and Oct4/Sox2^3bp^ complexes. (DOCX)

S4 Table Binding free energy of Oct4/Sox2^0bp^ and Oct4/Sox2^3bp^ complexes, as determined using g_mmpbsa (GROMACS tool). (DOCX)

## References

[1] Takahashi K, Tanabe K, Ohnuki M, Narita M, Ichisaka T, Tomoda K, et al Induction of pluripotent stem cells from adult human fibroblasts by defined factors. Cell. 2007; 131: 861–872. 18035408 10.1016/j.cell.2007.11.019

[2] Tantin D. Oct transcription factors in development and stem cells: insights and mechanisms. Development. 2013; 140: 2857–2866. 10.1242/dev.095927 23821033 PMC3699277

[3] Kamachi Y, Kondoh H. Sox proteins: regulators of cell fate specification and differentiation. Development. 2013; 140: 4129–4144. 10.1242/dev.091793 24086078

[4] Merino F, Ng CK, Veerapandian V, Scholer HR, Jauch R, Cojocaru V. Structural basis for the SOX-dependent genomic redistribution of OCT4 in stem cell differentiation. Structure. 2014; 22: 1274–1286. 10.1016/j.str.2014.06.014 25126959

[5] Jerabek S, Merino F, Scholer HR, Cojocaru V. OCT4: dynamic DNA binding pioneers stem cell pluripotency. Biochim Biophys Acta. 2014; 1839: 138–154. 10.1016/j.bbagrm.2013.10.001 24145198

[6] Pan GJ, Chang ZY, Scholer HR, Pei D. Stem cell pluripotency and transcription factor Oct4. Cell Res. 2002; 12: 321–329. 12528890 10.1038/sj.cr.7290134

[7] Esch D, Vahokoski J, Groves MR, Pogenberg V, Cojocaru V, Vom Bruch H, et al A unique Oct4 interface is crucial for reprogramming to pluripotency. Nat Cell Biol. 2013; 15: 295–301. 10.1038/ncb2680 23376973

[8] Kamachi Y, Uchikawa M, Kondoh H. Pairing SOX off: with partners in the regulation of embryonic development. Trends Genet. 2000; 16: 182–187. 10729834 10.1016/s0168-9525(99)01955-1

[9] Rodda DJ, Chew JL, Lim LH, Loh YH, Wang B, Ng HH, et al Transcriptional regulation of nanog by OCT4 and SOX2. J Biol Chem. 2005; 280: 24731–24737. 15860457 10.1074/jbc.M502573200

[10] van den Berg DL, Snoek T, Mullin NP, Yates A, Bezstarosti K, Demmers J, et al An Oct4-centered protein interaction network in embryonic stem cells. Cell Stem Cell. 2010; 6: 369–381. 10.1016/j.stem.2010.02.014 20362541 PMC2860243

[11] Remenyi A, Lins K, Nissen LJ, Reinbold R, Scholer HR, Wilmanns M. Crystal structure of a POU/HMG/DNA ternary complex suggests differential assembly of Oct4 and Sox2 on two enhancers. Genes Dev. 2003; 17: 2048–2059. 12923055 10.1101/gad.269303 PMC196258

[12] Williams DCJ, Cai M, Clore GM. Molecular basis for synergistic transcriptional activation by Oct1 and Sox2 revealed from the solution structure of the 42-kDa Oct1.Sox2.Hoxb1-DNA ternary transcription factor complex. J Biol Chem. 2004; 279: 1449–1457. 14559893 10.1074/jbc.M309790200

[13] Schmidt R, Plath K. The roles of the reprogramming factors Oct4, Sox2 and Klf4 in resetting the somatic cell epigenome during induced pluripotent stem cell generation. Genome Biol. 2012; 13: 251 10.1186/gb-2012-13-10-251 23088445 PMC3491406

[14] Karplus M, McCammon JA. Molecular dynamics simulations of biomolecules. Nat Struct Biol. 2002; 9: 646–652. 12198485 10.1038/nsb0902-646

[15] Chen L, Zheng QC, Zhang HX. Insights into the effects of mutations on Cren7-DNA binding using molecular dynamics simulations and free energy calculations. Phys Chem Chem Phys. 2015; 17: 5704–5711. 10.1039/c4cp05413j 25622968

[16] Govindaraj RG, Manavalan B, Lee G, Choi S. Molecular modeling-based evaluation of hTLR10 and identification of potential ligands in Toll-like receptor signaling. PLoS One. 2010; 5: e12713 10.1371/journal.pone.0012713 20877634 PMC2943521

[17] Manavalan B, Lee J, Lee J. Random forest-based protein model quality assessment (RFMQA) using structural features and potential energy terms. PLoS One. 2014; 9: e106542 10.1371/journal.pone.0106542 25222008 PMC4164442

[18] Kang J, Gemberling M, Nakamura M, Whitby FG, Handa H, Fairbrother WG, et al A general mechanism for transcription regulation by Oct1 and Oct4 in response to genotoxic and oxidative stress. Genes Dev. 2009; 23: 208–222. 10.1101/gad.1750709 19171782 PMC2648538

[19] Pettersen EF, Goddard TD, Huang CC, Couch GS, Greenblatt DM, Meng EC, et al UCSF Chimera—a visualization system for exploratory research and analysis. J Comput Chem. 2004; 25: 1605–1612. 15264254 10.1002/jcc.20084

[20] Pronk S, Pall S, Schulz R, Larsson P, Bjelkmar P, Apostolov R, et al GROMACS 4.5: a high-throughput and highly parallel open source molecular simulation toolkit. Bioinformatics. 2013; 29: 845–854. 10.1093/bioinformatics/btt055 23407358 PMC3605599

[21] Hornak V, Abel R, Okur A, Strockbine B, Roitberg A, Simmerling C. Comparison of multiple Amber force fields and development of improved protein backbone parameters. Proteins. 2006; 65: 712–725. 16981200 10.1002/prot.21123 PMC4805110

[22] Lindorff-Larsen K, Piana S, Palmo K, Maragakis P, Klepeis JL, Dror RO, et al Improved side-chain torsion potentials for the Amber ff99SB protein force field. Proteins. 2010; 78: 1950–1958. 10.1002/prot.22711 20408171 PMC2970904

[23] Hess B, Bekker H, Berendsen HJ, Fraaije JG. LINCS: a linear constraint solver for molecular simulations. J Comput Chem. 1997; 18: 1463–1472.

[24] Darden T, Perera L, Li L, Pedersen L. New tricks for modelers from the crystallography toolkit: the particle mesh Ewald algorithm and its use in nucleic acid simulations. Structure. 1999; 7: R55–60. 10368306 10.1016/s0969-2126(99)80033-1

[25] Maisuradze GG, Liwo A, Scheraga HA. Principal component analysis for protein folding dynamics. J Mol Biol. 2009; 385: 312–329. 10.1016/j.jmb.2008.10.018 18952103 PMC2652707

[26] Mesentean S, Fischer S, Smith JC. Analyzing large-scale structural change in proteins: comparison of principal component projection and Sammon mapping. Proteins. 2006; 64: 210–218. 16617427 10.1002/prot.20981

[27] Amadei A, Linssen AB, Berendsen HJ. Essential dynamics of proteins. Proteins. 1993; 17: 412–425. 8108382 10.1002/prot.340170408

[28] Upadhyay SK. Dynamics of Gal80p in the Gal80p-Gal3p complex differ significantly from the dynamics in the Gal80p-Gal1p complex: implications for the higher specificity of Gal3p. Mol Biosyst. 2014; 10: 3120–3129. 10.1039/c4mb00371c 25220841

[29] Chillemi G, D'Annessa I, Fiorani P, Losasso C, Benedetti P, Desideri A. Thr729 in human topoisomerase I modulates anti-cancer drug resistance by altering protein domain communications as suggested by molecular dynamics simulations. Nucleic Acids Res. 2008; 36: 5645–5651. 10.1093/nar/gkn558 18765473 PMC2553568

[30] Grant BJ, Rodrigues AP, ElSawy KM, McCammon JA, Caves LS. Bio3d: an R package for the comparative analysis of protein structures. Bioinformatics. 2006; 22: 2695–2696. 16940322 10.1093/bioinformatics/btl461

[31] Hou T, Wang J, Li Y, Wang W. Assessing the performance of the MM/PBSA and MM/GBSA methods. 1. The accuracy of binding free energy calculations based on molecular dynamics simulations. J Chem Inf Model. 2011; 51: 69–82. 10.1021/ci100275a 21117705 PMC3029230

[32] Miller BR 3rd, McGee TD Jr., Swails JM, Homeyer N, Gohlke H, Roitberg AE. MMPBSA.py: An Efficient Program for End-State Free Energy Calculations. J Chem Theory Comput. 2012; 8: 3314–3321. 10.1021/ct300418h 26605738

[33] Zoete V, Michielin O. Comparison between computational alanine scanning and per-residue binding free energy decomposition for protein-protein association using MM-GBSA: application to the TCR-p-MHC complex. Proteins. 2007; 67: 1026–1047. 17377991 10.1002/prot.21395

[34] Wang J, Wolf RM, Caldwell JW, Kollman PA, Case DA. Development and testing of a general amber force field. J Comput Chem. 2004; 25: 1157–1174. 15116359 10.1002/jcc.20035

[35] Lavery R, Moakher M, Maddocks JH, Petkeviciute D, Zakrzewska K. Conformational analysis of nucleic acids revisited: Curves+. Nucleic Acids Res. 2009; 37: 5917–5929. 10.1093/nar/gkp608 19625494 PMC2761274

[36] Boyer LA, Lee TI, Cole MF, Johnstone SE, Levine SS, Zucker JP, et al Core transcriptional regulatory circuitry in human embryonic stem cells. Cell. 2005; 122: 947–956. 16153702 10.1016/j.cell.2005.08.020 PMC3006442

[37] Yusuf D, Butland SL, Swanson MI, Bolotin E, Ticoll A, Cheung WA, et al The transcription factor encyclopedia. Genome Biol. 2012; 13: R24 10.1186/gb-2012-13-3-r24 22458515 PMC3439975

[38] Mathelier A, Zhao X, Zhang AW, Parcy F, Worsley-Hunt R, Arenillas DJ, et al JASPAR 2014: an extensively expanded and updated open-access database of transcription factor binding profiles. Nucleic Acids Res. 2014; 42: D142–147. 10.1093/nar/gkt997 24194598 PMC3965086

[39] Stothard P. The sequence manipulation suite: JavaScript programs for analyzing and formatting protein and DNA sequences. Biotechniques. 2000; 28: 1102, 1104. 10868275 10.2144/00286ir01

[40] Humphrey W, Dalke A, Schulten K. VMD: visual molecular dynamics. J Mol Graph. 1996; 14: 33–38, 27–38. 8744570 10.1016/0263-7855(96)00018-5

[41] Chen X, Xu H, Yuan P, Fang F, Huss M, Vega VB, et al Integration of external signaling pathways with the core transcriptional network in embryonic stem cells. Cell. 2008; 133: 1106–1117. 10.1016/j.cell.2008.04.043 18555785

[42] Lambrughi M, Papaleo E, Testa L, Brocca S, De Gioia L, Grandori R. Intramolecular interactions stabilizing compact conformations of the intrinsically disordered kinase-inhibitor domain of Sic1: a molecular dynamics investigation. Front Physiol. 2012; 3: 435 10.3389/fphys.2012.00435 23189058 PMC3504315

[43] Richmond TJ, Davey CA. The structure of DNA in the nucleosome core. Nature. 2003; 423: 145–150. 12736678 10.1038/nature01595

[44] Travers AA. DNA conformation and protein binding. Annu Rev Biochem. 1989; 58: 427–452. 2673015 10.1146/annurev.bi.58.070189.002235

[45] Rohs R, West SM, Sosinsky A, Liu P, Mann RS, Honig B. The role of DNA shape in protein-DNA recognition. Nature. 2009; 461: 1248–1253. 10.1038/nature08473 19865164 PMC2793086

[46] Palasingam P, Jauch R, Ng CK, Kolatkar PR. The structure of Sox17 bound to DNA reveals a conserved bending topology but selective protein interaction platforms. J Mol Biol. 2009; 388: 619–630. 10.1016/j.jmb.2009.03.055 19328208

[47] Vlahovicek K, Kajan L, Pongor S. DNA analysis servers: plot.it, bend.it, model.it and IS. Nucleic Acids Res. 2003; 31: 3686–3687. 12824394 10.1093/nar/gkg559 PMC168966

[48] Blanchet C, Pasi M, Zakrzewska K, Lavery R. CURVES+ web server for analyzing and visualizing the helical, backbone and groove parameters of nucleic acid structures. Nucleic Acids Res. 2011; 39: W68–73. 10.1093/nar/gkr316 21558323 PMC3125750

[49] Prive GG, Yanagi K, Dickerson RE. Structure of the B-DNA decamer C-C-A-A-C-G-T-T-G-G and comparison with isomorphous decamers C-C-A-A-G-A-T-T-G-G and C-C-A-G-G-C-C-T-G-G. J Mol Biol. 1991; 217: 177–199. 1988677 10.1016/0022-2836(91)90619-h

[50] Hays FA, Teegarden A, Jones ZJ, Harms M, Raup D, Watson J, et al How sequence defines structure: a crystallographic map of DNA structure and conformation. Proc Natl Acad Sci U S A. 2005; 102: 7157–7162. 15870206 10.1073/pnas.0409455102 PMC1129101

[51] Kumari R, Kumar R, Open Source Drug Discovery C, Lynn A. g_mmpbsa—a GROMACS tool for high-throughput MM-PBSA calculations. J Chem Inf Model. 2014; 54: 1951–1962. 10.1021/ci500020m 24850022

[52] Jauch R, Aksoy I, Hutchins AP, Ng CK, Tian XF, Chen J, et al Conversion of Sox17 into a pluripotency reprogramming factor by reengineering its association with Oct4 on DNA. Stem Cells. 2011; 29: 940–951. 10.1002/stem.639 21472822

[53] Scaffidi P, Bianchi ME. Spatially precise DNA bending is an essential activity of the sox2 transcription factor. J Biol Chem. 2001; 276: 47296–47302. 11584012 10.1074/jbc.M107619200

[54] Hilser VJ, Thompson EB. Structural dynamics, intrinsic disorder, and allostery in nuclear receptors as transcription factors. J Biol Chem. 2011; 286: 39675–39682. 10.1074/jbc.R111.278929 21937423 PMC3220581

[55] Manavalan B, Basith S, Choi YM, Lee G, Choi S. Structure-function relationship of cytoplasmic and nuclear IkappaB proteins: an in silico analysis. PLoS One. 2010; 5: e15782 10.1371/journal.pone.0015782 21203422 PMC3009747

[56] Pan Y, Nussinov R. p53-Induced DNA bending: the interplay between p53-DNA and p53-p53 interactions. J Phys Chem B. 2008; 112: 6716–6724. 10.1021/jp800680w 18461991 PMC2755056

[57] Lu Q, Tan YH, Luo R. Molecular dynamics simulations of p53 DNA-binding domain. J Phys Chem B. 2007; 111: 11538–11545. 17824689 10.1021/jp0742261 PMC2522240

[58] Bredenberg J, Nilsson L. Conformational states of the glucocorticoid receptor DNA-binding domain from molecular dynamics simulations. Proteins. 2002; 49: 24–36. 12211013 10.1002/prot.10195

[59] Merino F, Bouvier B, Cojocaru V. Cooperative DNA Recognition Modulated by an Interplay between Protein-Protein Interactions and DNA-Mediated Allostery. PLoS Comput Biol. 2015; 11: e1004287 10.1371/journal.pcbi.1004287 26067358 PMC4465831

