# Supplementary figures and images for: Structural Mechanism behind Distinct Efficiency of Oct4/Sox2 Proteins in Differentially Spaced DNA Complexes
Source: PLoS One. 2016 Jan 20;11(1):e0147240. doi: 10.1371/journal.pone.0147240 (PMC4720428; doi:10.1371/journal.pone.0147240)

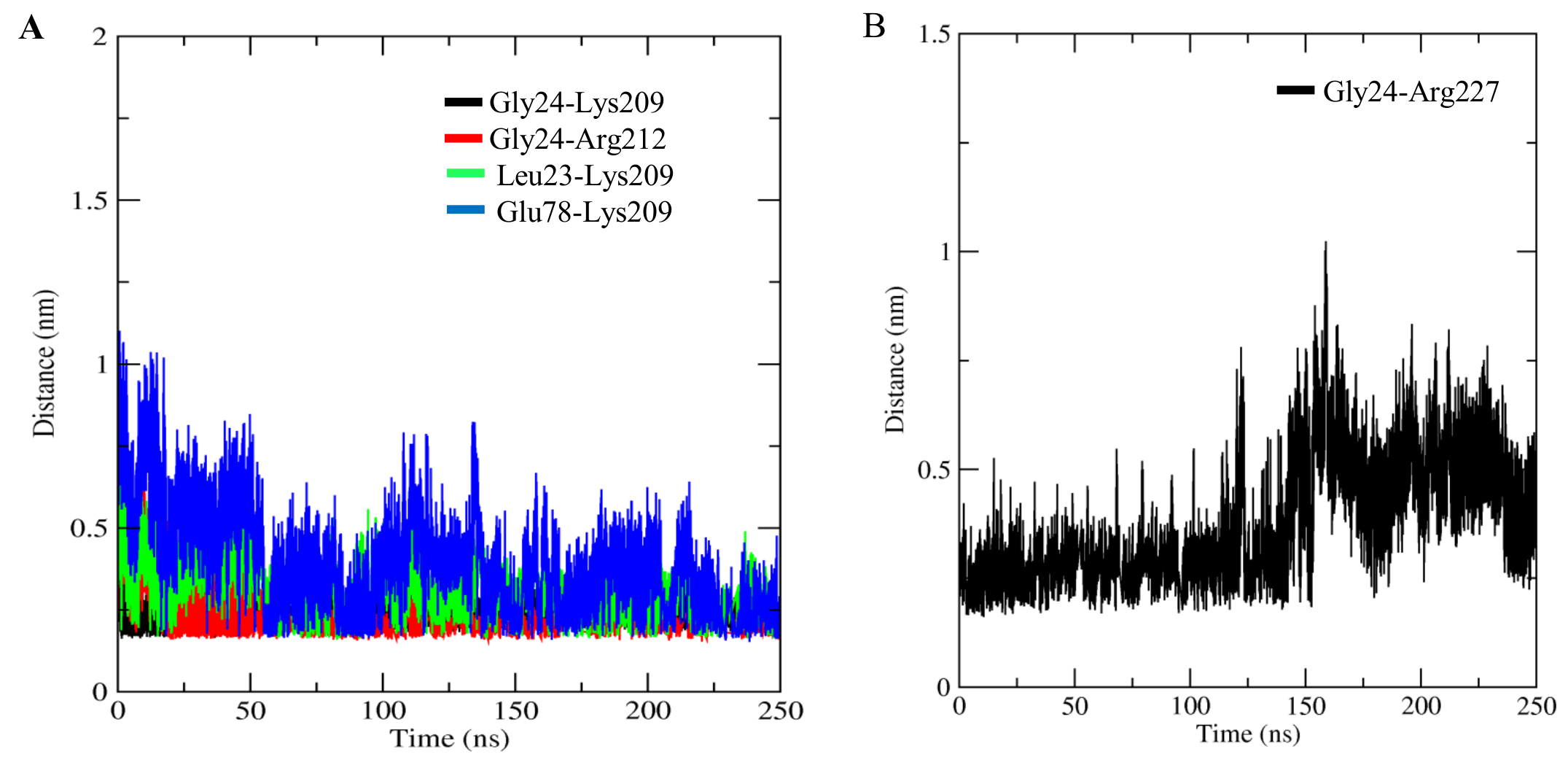

Supplement: S1 Fig — (A) Black represents the minimum distance between Gly24 of Oct4 and Lys209 of Sox2, red represents the minimum distance between Gly24 of Oct4 and Arg212 of Sox2, Green represents the minimum distance between Leu23 of Oct4 and Lys209 of Sox2, blue represent the minimum distance between Glu78 of Oct4 and Lys209 of Sox2 for the Oct4/Sox20bp complex. (B) The minimum distance between hydrogen bond-interacting residues (Gly24 of Oct4 and Arg227 of Sox2) for the Oct4/Sox23bp complex is indicated in black. (TIFF) [file pone.0147240.s001.tiff]

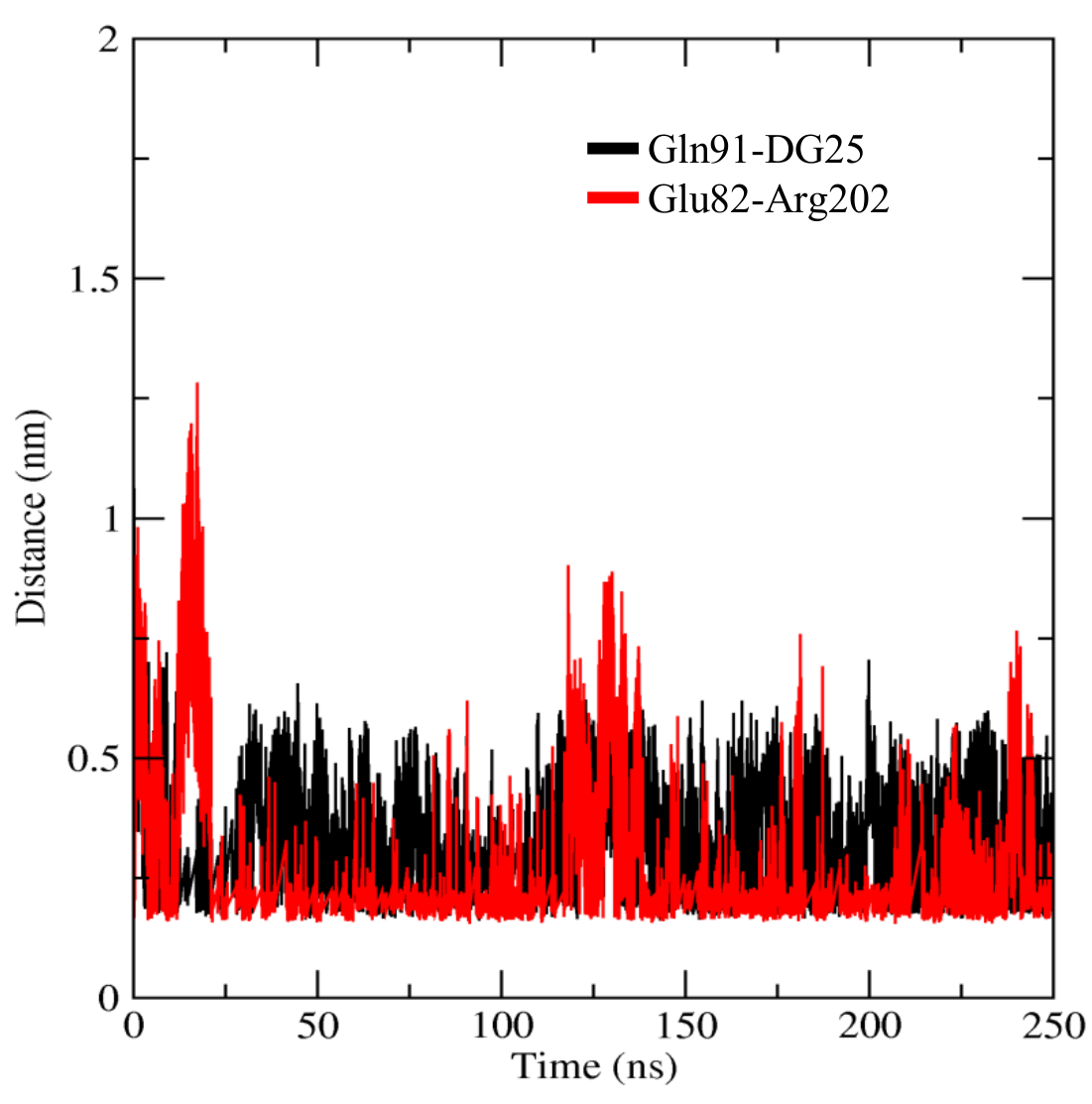

Supplement: S2 Fig — The minimum distances for hydrogen bond-interacting residues between Gln91 of the Oct4 linker and the DG25 nucleotide are shown in black. The minimum distances between the salt bridge-forming residues Gln82 (Oct4 linker) and Arg202 (Sox2) are shown in red. (TIFF) [file pone.0147240.s002.tiff]

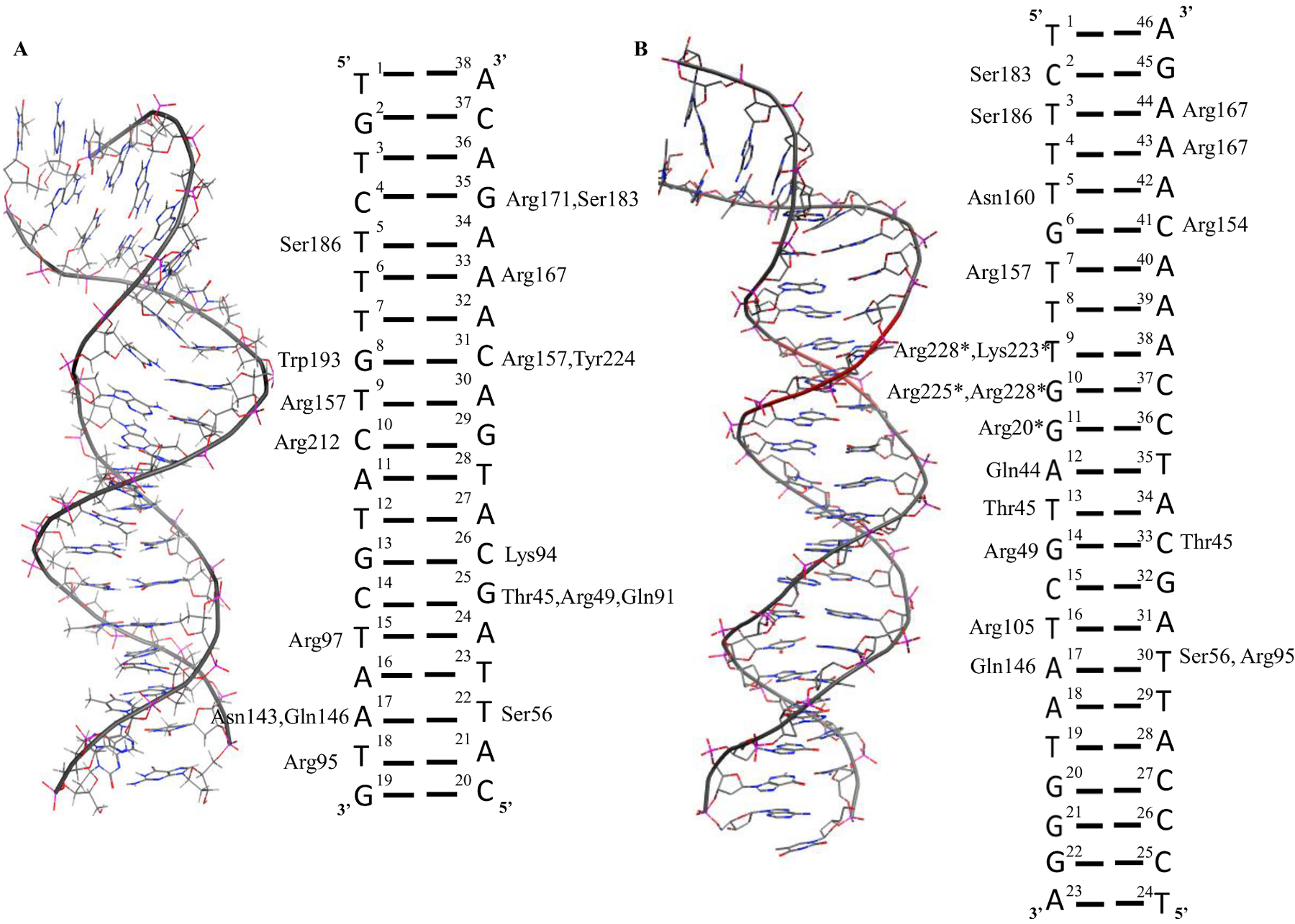

Supplement: S3 Fig — (A) Oct4 and Sox2 residues interacting with DNA in the Oct4/Sox20bp complex. (B) Oct4/Sox2 residues interacting with DNA in the Oct4/Sox23bp complex. Asterisks indicate residues interacting with DNA at the binding site separated by 3 base pairs (red-colored region). DNA is represented as a skeletal structure in grey, and prominently interacting residues are labeled. (TIFF) [file pone.0147240.s003.tiff]

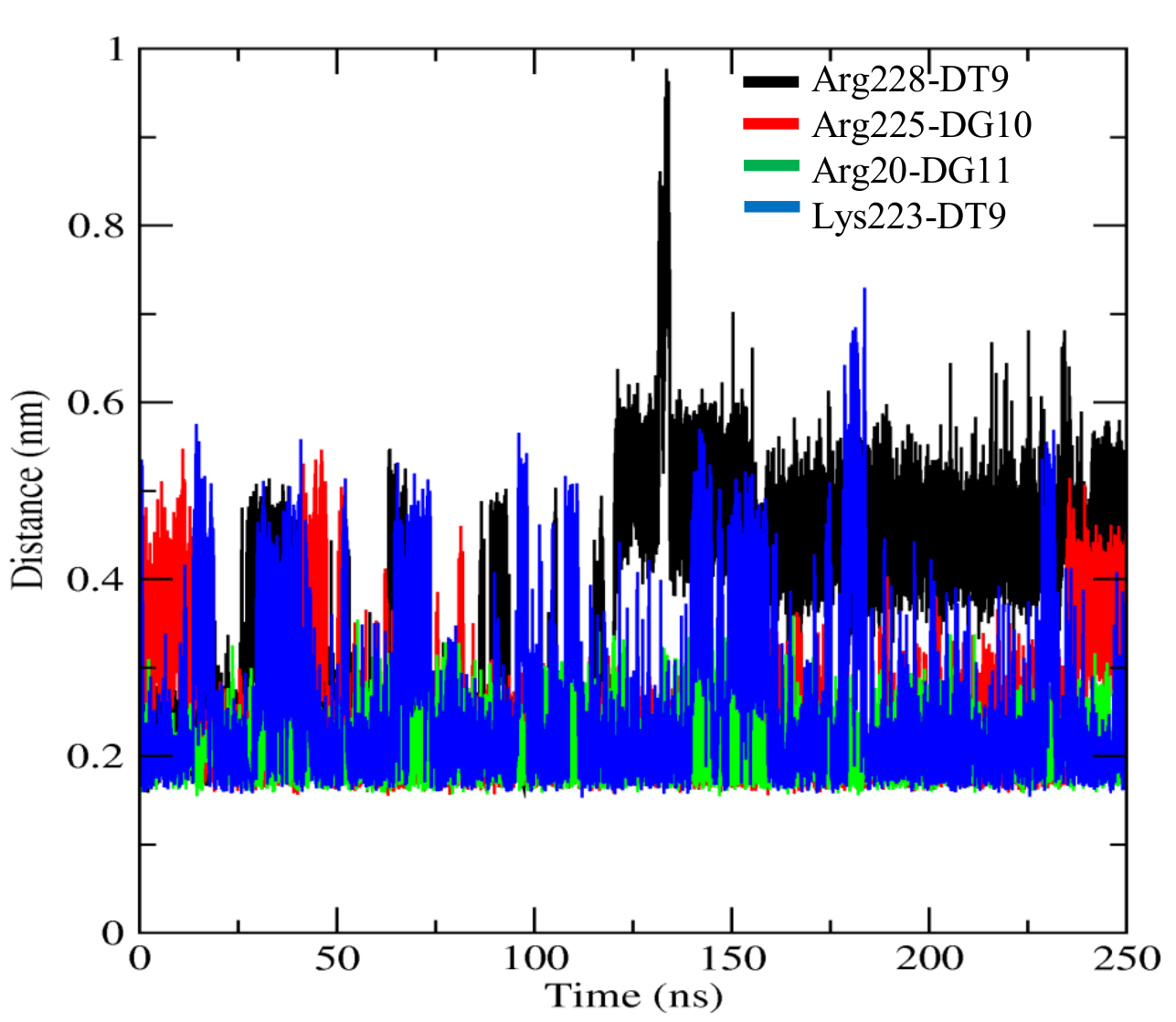

Supplement: S4 Fig — The minimum distance between hydrogen bond-interacting residues Lys223 (Sox2), Arg228 (Sox2); Arg225 (Sox2); Arg20 (Oct4); and DT9, DT9, DG10, and DG11 at the 3 base pairs-separated binding site for Oct4/Sox23bp are shown in blue, black, red, and green, respectively. (TIFF) [file pone.0147240.s004.tiff]

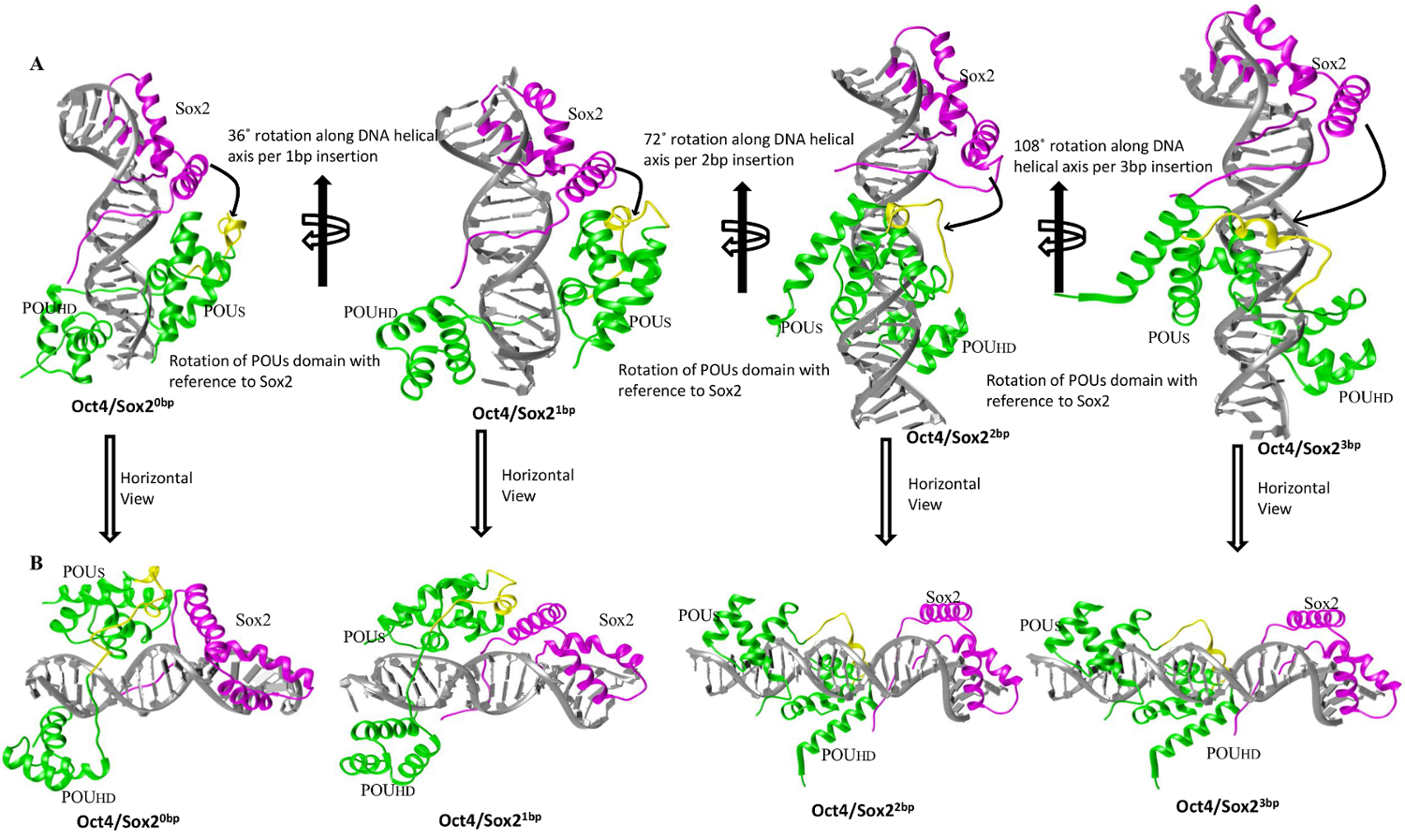

Supplement: S5 Fig — (A) Vertical representation of the Oct4 and Sox2 proteins with 0, 1, 2, or 3 base pairs insertions at their binding sites. (B) Horizontal representation of the Oct4 and Sox2 proteins with 0, 1, 2, or 3 base pairs insertions at their binding sites. The insertion of 1 base pair rotates the complex by 36° along the DNA helical axis, facilitating different rotational positioning of POUS with respect to the HMG box domain. The DNA is represented in grey, Sox2 is in magenta, and the Oct4 domains are in green. (TIFF) [file pone.0147240.s005.tiff]
