# Supplementary material for: Structural Mechanism behind Distinct Efficiency of Oct4/Sox2 Proteins in Differentially Spaced DNA Complexes
Source: PLoS One. 2016 Jan 20;11(1):e0147240. doi: 10.1371/journal.pone.0147240 (PMC4720428; doi:10.1371/journal.pone.0147240)
Supplement: S1 Table — (DOCX) [file pone.0147240.s006.docx]

**S1 Table. Differentially spaced Oct4 and Sox2-binding sites for target genes.**

| **No.** | **Target gene** | **Spacing between the binding site of Oct4 and Sox2** | **Organism** | **Function** | **Status** |
| --- | --- | --- | --- | --- | --- |
| 1 | *BHLHB5* | 0 | Human | Transcriptional repressor for Neurod1 and Myod-responsive genes | Predicted |
| 2 | *Fbxo15* | 0 | Mouse | Selection marker for pluripotent cells | Reported |
| 3 | *HOXB1* | 0 | Human | Developmental regulatory system | Reported |
| 4 | *Nanog* | 0 | Mouse | Proliferation and self-renewal | Reported |
| 5 | *Pou5f1* | 0 | Mouse | Controls embryonic development | Reported |
| 6 | *Smad1* | 0 | Mouse | Transcriptional modulator in spermatogenesis | Reported |
| 7 | *Sox2* | 0 | Mouse | Early embryogenesis, cell pluripotency | Reported |
| 8 | *Utf1* | 0 | Mouse | Transcriptional co-activator | Reported |
| 9 | *Lefty1* | 0 | Mouse | Regulator of LEFTY2 and NODAL binding | Reported |
| 10 | *Dppa4* | 1 | Mouse | Involved in epigenetic regulation | Reported |
| 11 | *Dc5* | 1 | Mouse | Involved in the initiation of DNA replication | Reported |
| 12 | *TBX5* | 1 | Human | Transcriptional regulation of genes required for mesoderm differentiation. | Predicted |
| 13 | *SYNPR* | 1 | Human | Intrinsic membrane protein of small synaptic vesicles. | Predicted |
| 14 | *SOX5* | 1 | Human | Activates transcription of COL2A1 and AGC1 | Predicted |
| 15 | *ATBF1* | 3 | Human | Regulator of myoblast differentiation | Predicted |
| 16 | *Fgf4* | 3 | Mouse | Embryonic development, proliferation, and differentiation | Reported |
| 17 | *FOXD3* | 3 | Human | Maintain pluripotent cells in the pre-implantation and peri-implantation stages of embryogenesis | Predicted |
| 18 | *SHC3* | 3 | Human | Adapter in Neuron Signaling | Predicted |
| 19 | *SPI-C* | 3 | Human | Development of red pulp macrophages | Predicted |
| 20 | *TLE3* | 3 | Human | Transcriptional co-repressor in Wnt signaling | Predicted |
| 21 | *TITF1* | 3 | Human | Maintenance of the thyroid differentiation | Predicted |
| 22 | *USP44* | 3 | Human | Regulatory role in the mitotic checkpoint | Predicted |
