# Supplementary material for: Structural Mechanism behind Distinct Efficiency of Oct4/Sox2 Proteins in Differentially Spaced DNA Complexes
Source: PLoS One. 2016 Jan 20;11(1):e0147240. doi: 10.1371/journal.pone.0147240 (PMC4720428; doi:10.1371/journal.pone.0147240)
Supplement: S2 Table — (DOCX) [file pone.0147240.s007.docx]

**S2 Table.** **Hydrogen bond interactions between DNA and Oct4/Sox2 complexes.**

| **Oct4/Sox2^0bp^** | **Nucleotide** | **Hydrogen bond description** | **Interacting residues** |
| --- | --- | --- | --- |
| Sox2 | DT5 | O2 .OG | Ser186 |
|  | DT6 | O2 .NH2 | Arg170 |
|  | DT6 | O1P .NZ | Lys187 |
|  | DT7 | O2 .ND2 | Asn160 |
|  | DG8 | O1P .NE1 | Trp193 |
|  | DT9 | O2 .NH2 | Arg157 |
|  | DT12 | O1P .NH2 | Arg228 |
|  | DG13 | O1P .NZ | Lys231 |
|  | DC14 | O1P .OG1 | Thr232 |
|  | DT28 | O3* .NZ | Lys229 |
|  | DG29 | O1P .NZ | Lys229 |
|  | DA30 | O2P .NH2 | Arg227 |
|  | DC31 | O2 .NH1 | Arg157 |
|  | DC31 | O4* .OH | Tyr224 |
|  | DC31 | O2P .NE | Arg227 |
|  | DA32 | O1P .NH2 | Arg154 |
|  | DA33 | O1P .NZ | Lys156 |
|  | DA33 | O3* .NE | Arg167 |
|  | DA34 | O1P .NH2 | Arg167 |
|  | DG35 | O1P .NE | Arg171 |
|  | DC10 | O4* .NH2 | Arg157 |
|  | DC10 | O1P .NH2 | Arg212 |
|  | DG35 | N2 .OG | Ser183 |
|  | DA11 | O1P .NZ | Lys223 |
| Oct4 | DA11 | O2P .NZ | Lys17 |
|  | DC10 | O1P .NH1 | Arg20 |
|  | DG13 | O6 .NH2 | Arg49 |
|  | DT18 | O4* .NH1 | Arg95 |
|  | DT15 | O4* .NH2 | Arg97 |
|  | DA17 | O1P .OG1 | Thr98 |
|  | DA16 | O2P .ND2 | Asn143 |
|  | DA17 | N6 .OD1 | Asn143 |
|  | DA17 | N6 .OE1 | Gln146 |
|  | DT15 | O2P .NZ | Lys147 |
|  | DC14 | O2P .NH1 | Arg150 |
|  | DG25 | O2P .OG | Ser43 |
|  | DG25 | O2P .OG1 | Thr45 |
|  | DG25 | O6 .NH2 | Arg49 |
|  | DT22 | O3* .OG | Ser56 |
|  | DT23 | O1P .OG | Ser56 |
|  | DT23 | O1P .ND2 | Asn59 |
|  | DG25 | O1P .NE2 | Gln91 |
|  | DC26 | O2P .NZ | Lys94 |
|  | DC26 | O1P .NZ | Lys96 |
|  | DT22 | O4 .NE2 | Gln146 |
| **Oct4/Sox2^3bp^** | **Nucleotide** | **Hydrogen bond description** | **Interacting residues** |
| Sox2 | DC2 | O3* .OG | Ser183 |
|  | DT3 | O2 .OG | Ser186 |
|  | DT3 | O3* .NZ | Lys187 |
|  | DT4 | O1P .NZ | Lys187 |
|  | DT5 | O2 .ND2 | Asn160 |
|  | DT5 | O3* .NE1 | Trp193 |
|  | DG6 | O1P .NE1 | Trp193 |
|  | DT7 | O2 .NH1 | Arg157 |
|  | DT8 | O4* .NH2 | Arg157 |
|  | DT9 | O1P .NZ | Lys223 |
|  | DT9 | O2 .NH2 | Arg228 |
|  | DG10 | O1P .NE | Arg225 |
|  | DA38 | O3* .NZ | Lys231 |
|  | DA39 | O4* .NH1 | Arg228 |
|  | DC41 | O1P .NH2 | Arg154 |
|  | DC41 | O2 .NH1 | Arg157 |
|  | DC41 | O4* .OH | Tyr224 |
|  | DA43 | O3* .NE | Arg167 |
|  | DA44 | O1P .NH2 | Arg167 |
|  | DG45 | O2P .NH2 | Arg171 |
|  | DT1 | O2 .NE2 | His181 |
|  | DT8 | O1P .NH2 | Arg212 |
|  | DA42 | O2P .NH2 | Arg154 |
|  | DA43 | O1P .NZ | Lys156 |
|  | DG45 | O2P .NH2 | Arg171 |
|  | DG45 | O4* .ND2 | Asn182 |
|  | DG10 | O4* .NE | Arg228 |
| Oct4 | DG11 | O1P .NH1 | Arg20 |
|  | DA12 | O1P .NZ | Lys17 |
|  | DA12 | N6 .OE1 | Gln44 |
|  | DT13 | O4 .OG1 | Thr45 |
|  | DG14 | N7 .NH2 | Arg49 |
|  | DT16 | O4* .NH2 | Arg97 |
|  | DT16 | O1P .NH1 | Arg105 |
|  | DT16 | O2P .NZ | Lys147 |
|  | DA17 | O4* .NE | Arg97 |
|  | DA17 | O2P .ND2 | Asn143 |
|  | DA17 | N6 .OE1 | Gln146 |
|  | DA18 | O1P .OG1 | Thr98 |
|  | DC26 | O2P .NH2 | Arg145 |
|  | DT30 | O2P .OG | Ser56 |
|  | DT30 | O2 .NH2 | Arg95 |
|  | DA31 | O2P .NZ | Lys40 |
|  | DG32 | O2P .OG | Ser43 |
|  | DG32 | O2P .OG1 | Thr46 |
|  | DC33 | N4 .OG1 | Thr45 |
|  | DC33 | O2P .NZ | Lys94 |
