# Supplementary material for: Structural Mechanism behind Distinct Efficiency of Oct4/Sox2 Proteins in Differentially Spaced DNA Complexes
Source: PLoS One. 2016 Jan 20;11(1):e0147240. doi: 10.1371/journal.pone.0147240 (PMC4720428; doi:10.1371/journal.pone.0147240)
Supplement: S3 Table — (DOCX) [file pone.0147240.s008.docx]

**S3 Table. Relative binding free energy for residues** **of the Oct4/Sox2^0bp^ and Oct4/Sox2^3bp^ complexes.**

| **Oct4/Sox2^0bp^ (kcal mol^-1^)** | | | | **Oct4/Sox2^3bp^ (kcal mol^-1^)** | | | |
| --- | --- | --- | --- | --- | --- | --- | --- |
| **Residues** | **Decomposition energy** | **Residues** | **Decomposition energy** | **Residues** | **Decomposition energy** | **Residues** | **Decomposition energy** |
| ASP 1 | 0.195 +/- 0.012 | LYS 117 | -0.026 +/- 0.037 | ASP 1 | 0.027 +/- 0.002 | LYS 117 | -6.328 +/- 1.261 |
| ILE 2 | -0.015 +/- 0.002 | PRO 118 | 0.042 +/- 0.012 | ILE 2 | -0.002 +/- 0.001 | PRO 118 | 0.137 +/- 0.015 |
| LYS 3 | -0.235 +/- 0.004 | THR 119 | -0.004 +/- 0.006 | LYS 3 | -0.169 +/- 0.002 | THR 119 | -0.036 +/- 0.013 |
| ALA 4 | 0.014 +/- 0.001 | LEU 120 | -0.001 +/- 0.008 | ALA 4 | 0.001 +/- 0.002 | LEU 120 | -0.685 +/- 0.094 |
| LEU 5 | 0.008 +/- 0.004 | GLN 121 | 0.030 +/- 0.002 | LEU 5 | -0.036 +/- 0.006 | GLN 121 | 0.046 +/- 0.004 |
| GLN 6 | 0.095 +/- 0.012 | GLN 122 | 0.050 +/- 0.003 | GLN 6 | 0.013 +/- 0.013 | GLN 122 | 0.031 +/- 0.009 |
| LYS 7 | -0.191 +/- 0.001 | ILE 123 | -0.018 +/- 0.013 | LYS 7 | -0.272 +/- 0.005 | ILE 123 | -0.341 +/- 0.046 |
| GLU 8 | 0.235 +/- 0.005 | SER 124 | 0.023 +/- 0.008 | GLU 8 | 0.345 +/- 0.005 | SER 124 | 0.096 +/- 0.017 |
| LEU 9 | 0.008 +/- 0.013 | HIE 125 | 0.006 +/- 0.002 | LEU 9 | -0.051 +/- 0.009 | HIE 125 | 0.007 +/- 0.003 |
| GLU 10 | 0.469 +/- 0.007 | ILE 126 | -0.031 +/- 0.014 | GLU 10 | 0.443 +/- 0.012 | ILE 126 | -0.025 +/- 0.011 |
| GLN 11 | 0.030 +/- 0.008 | ALA 127 | 0.010 +/- 0.007 | GLN 11 | 0.036 +/- 0.004 | ALA 127 | 0.025 +/- 0.007 |
| PHE 12 | -0.036 +/- 0.008 | GLN 128 | 0.014 +/- 0.005 | PHE 12 | -0.128 +/- 0.010 | GLN 128 | 0.037 +/- 0.003 |
| ALA 13 | -0.044 +/- 0.020 | GLN 129 | 0.027 +/- 0.003 | ALA 13 | -0.063 +/- 0.019 | GLN 129 | 0.054 +/- 0.002 |
| LYS 14 | 0.197 +/- 0.058 | LEU 130 | 0.010 +/- 0.003 | LYS 14 | -0.318 +/- 0.013 | LEU 130 | 0.022 +/- 0.003 |
| LEU 15 | -0.013 +/- 0.023 | GLY 131 | 0.034 +/- 0.004 | LEU 15 | -0.036 +/- 0.012 | GLY 131 | 0.041 +/- 0.003 |
| LEU 16 | -0.201 +/- 0.040 | LEU 132 | -0.062 +/- 0.019 | LEU 16 | -0.322 +/- 0.038 | LEU 132 | 0.011 +/- 0.007 |
| LYS 17 | -7.966 +/- 0.416 | GLU 133 | 0.461 +/- 0.060 | LYS 17 | -8.610 +/- 0.882 | GLU 133 | 0.428 +/- 0.018 |
| GLN 18 | 0.082 +/- 0.029 | LYS 134 | -0.394 +/- 0.045 | GLN 18 | 0.128 +/- 0.013 | LYS 134 | -0.655 +/- 0.073 |
| LYS 19 | -0.259 +/- 0.027 | ASP 135 | 0.614 +/- 0.199 | LYS 19 | -0.330 +/- 0.021 | ASP 135 | 0.982 +/- 0.071 |
| ARG 20 | -11.027 +/- 0.46 | VAL 136 | -2.571 +/- 0.307 | ARG 20 | -11.163 +/- 0.718 | VAL 136 | -0.623 +/- 0.061 |
| ILE 21 | -0.753 +/- 0.040 | VAL 137 | -0.002 +/- 0.074 | ILE 21 | 0.068 +/- 0.074 | VAL 137 | -0.509 +/- 0.072 |
| THR 22 | 0.235 +/- 0.035 | ARG 138 | -0.676 +/- 0.121 | THR 22 | 0.130 +/- 0.013 | ARG 138 | -7.390 +/- 1.345 |
| LEU 23 | -0.040 +/- 0.032 | VAL 139 | -4.284 +/- 0.244 | LEU 23 | 0.082 +/- 0.017 | VAL 139 | -2.744 +/- 0.258 |
| GLY 24 | -0.230 +/- 0.058 | TRP 140 | -1.033 +/- 0.131 | GLY 24 | -0.065 +/- 0.034 | TRP 140 | -2.089 +/- 0.626 |
| TYR 25 | -0.796 +/- 0.196 | PHE 141 | 0.060 +/- 0.070 | TYR 25 | -0.625 +/- 0.111 | PHE 141 | -0.408 +/- 0.075 |
| THR 26 | -8.373 +/- 0.563 | CYS 142 | -1.040 +/- 0.416 | THR 26 | -6.312 +/- 0.413 | CYS 142 | -0.668 +/- 0.597 |
| GLN 27 | -7.460 +/- 0.951 | ASN 143 | 12.623 +/- 1.443 | GLN 27 | -10.047 +/- 0.642 | ASN 143 | -4.176 +/- 0.365 |
| ALA 28 | -0.626 +/- 0.143 | ARG 144 | -0.756 +/- 0.193 | ALA 28 | -0.358 +/- 0.179 | ARG 144 | -1.264 +/- 0.220 |
| ASP 29 | 2.768 +/- 0.422 | ARG 145 | 0.446 +/- 0.139 | ASP 29 | 1.535 +/- 0.094 | ARG 145 | 13.479 +/- 1.286 |
| VAL 30 | -0.491 +/- 0.042 | GLN 146 | -0.087 +/- 0.460 | VAL 30 | -1.322 +/- 0.139 | GLN 146 | -3.381 +/- 0.810 |
| GLY 31 | 0.044 +/- 0.016 | LYS 147 | -2.575 +/- 0.648 | GLY 31 | -0.131 +/- 0.060 | LYS 147 | -9.881 +/- 1.118 |
| LEU 32 | -0.020 +/- 0.063 | GLY 148 | 0.109 +/- 0.010 | LEU 32 | -0.168 +/- 0.051 | GLY 148 | -1.138 +/- 0.903 |
| THR 33 | 0.002 +/- 0.027 | LYS 149 | -0.206 +/- 0.008 | THR 33 | -0.052 +/- 0.030 | LYS 149 | -0.172 +/- 0.029 |
| LEU 34 | -0.083 +/- 0.029 | ARG 150 | -0.160 +/- 0.002 | LEU 34 | -0.334 +/- 0.057 | ARG 150 | -4.728 +/- 0.979 |
| GLY 35 | 0.095 +/- 0.006 | SER 151 | 0.028 +/- 0.002 | GLY 35 | 0.186 +/- 0.021 | SER 151 | 0.138 +/- 0.017 |
| VAL 36 | 0.026 +/- 0.003 | SER 152 | 0.210 +/- 0.005 | VAL 36 | -0.013 +/- 0.028 | SER 152 | 0.701 +/- 0.030 |
| LEU 37 | 0.035 +/- 0.002 | ASP 153 | 0.276 +/- 0.012 | LEU 37 | -0.010 +/- 0.008 | ASP 153 | 0.181 +/- 0.028 |
| PHE 38 | 0.026 +/- 0.003 | ARG 154 | 11.695 +/- 0.962 | PHE 38 | -0.023 +/- 0.006 | ARG 154 | 12.344 +/- 1.519 |
| GLY 39 | 0.040 +/- 0.003 | VAL 155 | -0.028 +/- 0.114 | GLY 39 | -0.012 +/- 0.013 | VAL 155 | -0.373 +/- 0.068 |
| LYS 40 | -0.665 +/- 0.140 | LYS 156 | -7.952 +/- 0.801 | LYS 40 | -14.391 +/- 0.799 | LYS 156 | 11.992 +/- 0.818 |
| VAL 41 | -0.394 +/- 0.048 | ARG 157 | 13.229 +/- 1.146 | VAL 41 | -0.880 +/- 0.223 | ARG 157 | 13.137 +/- 0.934 |
| PHE 42 | -1.076 +/- 0.084 | PRO 158 | 0.094 +/- 0.146 | PHE 42 | -5.194 +/- 0.318 | PRO 158 | -0.496 +/- 0.107 |
| SER 43 | -3.784 +/- 0.603 | MET 159 | -2.331 +/- 0.183 | SER 43 | -5.735 +/- 0.455 | MET 159 | -1.841 +/- 0.153 |
| GLN 44 | -1.979 +/- 0.231 | ASN 160 | -4.822 +/- 1.059 | GLN 44 | -4.361 +/- 0.741 | ASN 160 | -6.185 +/- 0.369 |
| THR 45 | -3.402 +/- 0.328 | ALA 161 | -1.389 +/- 0.200 | THR 45 | -5.113 +/- 0.490 | ALA 161 | -0.833 +/- 0.130 |
| THR 46 | -2.680 +/- 0.683 | PHE 162 | -4.935 +/- 0.192 | THR 46 | -3.922 +/- 0.456 | PHE 162 | -5.024 +/- 0.233 |
| ILE 47 | -1.236 +/- 0.165 | MET 163 | -5.881 +/- 0.196 | ILE 47 | -0.945 +/- 0.141 | MET 163 | -5.112 +/- 0.256 |
| CYS 48 | -1.318 +/- 0.124 | VAL 164 | -0.778 +/- 0.089 | CYS 48 | -1.995 +/- 0.572 | VAL 164 | -0.625 +/- 0.109 |
| ARG 49 | -4.493 +/- 0.690 | TRP 165 | -0.249 +/- 0.057 | ARG 49 | -5.739 +/- 1.012 | TRP 165 | -0.213 +/- 0.049 |
| PHE 50 | -0.471 +/- 0.105 | SER 166 | -0.695 +/- 0.120 | PHE 50 | -0.468 +/- 0.094 | SER 166 | -0.862 +/- 0.087 |
| GLU 51 | 2.329 +/- 0.326 | ARG 167 | -8.845 +/- 0.297 | GLU 51 | 2.882 +/- 0.498 | ARG 167 | -9.528 +/- 0.540 |
| ALA 52 | -0.237 +/- 0.122 | GLY 168 | 0.095 +/- 0.039 | ALA 52 | -0.347 +/- 0.056 | GLY 168 | -0.034 +/- 0.021 |
| LEU 53 | 0.238 +/- 0.057 | GLN 169 | -0.364 +/- 0.126 | LEU 53 | 0.133 +/- 0.027 | GLN 169 | -0.244 +/- 0.037 |
| GLN 54 | 0.173 +/- 0.118 | ARG 170 | -9.019 +/- 1.011 | GLN 54 | 0.407 +/- 0.472 | ARG 170 | -4.472 +/- 1.503 |
| LEU 55 | -6.907 +/- 0.417 | ARG 171 | 14.425 +/- 0.659 | LEU 55 | -1.238 +/- 0.121 | ARG 171 | -8.424 +/- 0.818 |
| SER 56 | -5.060 +/- 0.597 | LYS 172 | -0.136 +/- 0.054 | SER 56 | -0.659 +/- 0.414 | LYS 172 | -0.090 +/- 0.028 |
| PHE 57 | -0.049 +/- 0.020 | MET 173 | -0.277 +/- 0.076 | PHE 57 | -0.195 +/- 0.050 | MET 173 | -0.103 +/- 0.071 |
| LYS 58 | -0.846 +/- 0.028 | ALA 174 | -0.537 +/- 0.117 | LYS 58 | -8.517 +/- 0.447 | ALA 174 | -0.225 +/- 0.209 |
| ASN 59 | -7.520 +/- 0.982 | GLN 175 | 0.200 +/- 0.024 | ASN 59 | -4.470 +/- 0.659 | GLN 175 | 0.132 +/- 0.026 |
| MET 60 | -0.094 +/- 0.054 | GLU 176 | 0.434 +/- 0.011 | MET 60 | 0.004 +/- 0.062 | GLU 176 | 0.368 +/- 0.016 |
| CYS 61 | 0.020 +/- 0.047 | ASN 177 | 0.073 +/- 0.020 | CYS 61 | 0.012 +/- 0.041 | ASN 177 | 0.235 +/- 0.019 |
| LYS 62 | -0.340 +/- 0.075 | PRO 178 | -0.190 +/- 0.065 | LYS 62 | -1.481 +/- 0.153 | PRO 178 | 0.016 +/- 0.008 |
| LEU 63 | -0.416 +/- 0.021 | LYS 179 | -5.928 +/- 0.767 | LEU 63 | -0.454 +/- 0.070 | LYS 179 | -0.073 +/- 0.035 |
| ARG 64 | -0.150 +/- 0.016 | MET 180 | -0.878 +/- 0.183 | ARG 64 | -0.253 +/- 0.024 | MET 180 | -0.355 +/- 0.152 |
| PRO 65 | 0.023 +/- 0.009 | HIE 181 | -5.287 +/- 0.320 | PRO 65 | 0.031 +/- 0.007 | HIE 181 | -4.988 +/- 0.377 |
| LEU 66 | 0.001 +/- 0.017 | ASN 182 | -6.435 +/- 0.823 | LEU 66 | -0.111 +/- 0.025 | ASN 182 | -6.624 +/- 0.543 |
| LEU 67 | -0.063 +/- 0.018 | SER 183 | -3.061 +/- 0.432 | LEU 67 | -0.090 +/- 0.019 | SER 183 | -2.895 +/- 0.403 |
| GLN 68 | 0.045 +/- 0.005 | GLU 184 | 0.671 +/- 0.119 | GLN 68 | 0.058 +/- 0.007 | GLU 184 | 0.408 +/- 0.094 |
| LYS 69 | -0.151 +/- 0.006 | ILE 185 | -1.230 +/- 0.229 | LYS 69 | -0.244 +/- 0.006 | ILE 185 | -0.633 +/- 0.176 |
| TRP 70 | -0.158 +/- 0.021 | SER 186 | -1.366 +/- 0.458 | TRP 70 | -0.090 +/- 0.010 | SER 186 | -1.196 +/- 0.688 |
| VAL 71 | 0.068 +/- 0.010 | LYS 187 | -1.441 +/- 0.594 | VAL 71 | 0.060 +/- 0.008 | LYS 187 | -4.274 +/- 0.367 |
| GLU 72 | 0.156 +/- 0.005 | ARG 188 | -0.499 +/- 0.072 | GLU 72 | 0.234 +/- 0.004 | ARG 188 | -0.583 +/- 0.094 |
| GLU 73 | 0.166 +/- 0.003 | LEU 189 | -0.983 +/- 0.097 | GLU 73 | 0.251 +/- 0.004 | LEU 189 | -1.143 +/- 0.116 |
| ALA 74 | 0.048 +/- 0.007 | GLY 190 | -2.048 +/- 0.062 | ALA 74 | 0.026 +/- 0.004 | GLY 190 | -1.609 +/- 0.195 |
| ASP 75 | 0.184 +/- 0.006 | ALA 191 | 0.034 +/- 0.087 | ASP 75 | 0.287 +/- 0.004 | ALA 191 | 0.070 +/- 0.053 |
| ASN 76 | 0.015 +/- 0.004 | GLU 192 | 0.300 +/- 0.053 | ASN 76 | 0.004 +/- 0.003 | GLU 192 | 0.273 +/- 0.032 |
| ASN 77 | 0.039 +/- 0.001 | TRP 193 | -4.689 +/- 0.268 | ASN 77 | 0.016 +/- 0.003 | TRP 193 | -5.051 +/- 0.293 |
| GLU 78 | 0.313 +/- 0.011 | LYS 194 | -0.430 +/- 0.038 | GLU 78 | 0.448 +/- 0.011 | LYS 194 | -1.371 +/- 0.133 |
| ASN 79 | 0.035 +/- 0.017 | LEU 195 | -0.027 +/- 0.007 | ASN 79 | 0.003 +/- 0.004 | LEU 195 | -0.031 +/- 0.021 |
| LEU 80 | 0.018 +/- 0.011 | LEU 196 | -0.101 +/- 0.018 | LEU 80 | -0.040 +/- 0.013 | LEU 196 | -0.061 +/- 0.017 |
| GLN 81 | 0.030 +/- 0.018 | SER 197 | 0.032 +/- 0.009 | GLN 81 | 0.029 +/- 0.015 | SER 197 | 0.111 +/- 0.016 |
| GLU 82 | 0.520 +/- 0.016 | GLU 198 | 0.392 +/- 0.020 | GLU 82 | 0.572 +/- 0.015 | GLU 198 | 0.532 +/- 0.018 |
| ILE 83 | 0.005 +/- 0.003 | THR 199 | 0.038 +/- 0.004 | ILE 83 | 0.018 +/- 0.005 | THR 199 | 0.021 +/- 0.003 |
| CYS 84 | 0.014 +/- 0.003 | GLU 200 | 0.289 +/- 0.010 | CYS 84 | 0.039 +/- 0.014 | GLU 200 | 0.225 +/- 0.016 |
| LYS 85 | -0.241 +/- 0.003 | LYS 201 | -1.677 +/- 0.127 | LYS 85 | -0.548 +/- 0.032 | LYS 201 | -1.736 +/- 0.167 |
| ALA 86 | 0.014 +/- 0.003 | ARG 202 | -0.339 +/- 0.020 | ALA 86 | 0.035 +/- 0.007 | ARG 202 | -0.162 +/- 0.015 |
| GLU 87 | 0.311 +/- 0.010 | PRO 203 | 0.007 +/- 0.016 | GLU 87 | 0.620 +/- 0.020 | PRO 203 | -0.013 +/- 0.008 |
| THR 88 | 0.042 +/- 0.006 | PHE 204 | -0.340 +/- 0.083 | THR 88 | 0.012 +/- 0.002 | PHE 204 | -0.222 +/- 0.030 |
| LEU 89 | 0.062 +/- 0.008 | ILE 205 | -0.313 +/- 0.051 | LEU 89 | 0.035 +/- 0.004 | ILE 205 | 0.010 +/- 0.032 |
| VAL 90 | -0.062 +/- 0.019 | ASP 206 | 0.365 +/- 0.030 | VAL 90 | 0.026 +/- 0.005 | ASP 206 | 0.414 +/- 0.025 |
| GLN 91 | 0.198 +/- 0.426 | GLU 207 | 0.217 +/- 0.044 | GLN 91 | 0.177 +/- 0.020 | GLU 207 | 0.275 +/- 0.028 |
| ALA 92 | 0.094 +/- 0.019 | ALA 208 | -0.732 +/- 0.128 | ALA 92 | 0.041 +/- 0.052 | ALA 208 | -0.258 +/- 0.054 |
| ARG 93 | -0.407 +/- 0.026 | LYS 209 | 0.136 +/- 0.055 | ARG 93 | -9.178 +/- 1.044 | LYS 209 | -0.252 +/- 0.026 |
| LYS 94 | -8.011 +/- 0.405 | ARG 210 | -0.228 +/- 0.030 | LYS 94 | -2.548 +/- 0.423 | ARG 210 | -0.260 +/- 0.025 |
| ARG 95 | -11.710 +/- 1.036 | LEU 211 | -0.173 +/- 0.042 | ARG 95 | -16.835 +/- 1.354 | LEU 211 | -0.162 +/- 0.031 |
| LYS 96 | -5.051 +/- 0.506 | ARG 212 | -7.048 +/- 0.916 | LYS 96 | -0.511 +/- 0.314 | ARG 212 | -7.810 +/- 0.428 |
| ARG 97 | -17.853 +/- 1.520 | ALA 213 | 0.104 +/- 0.009 | ARG 97 | -11.685 +/- 1.045 | ALA 213 | 0.070 +/- 0.010 |
| THR 98 | -3.392 +/- 0.382 | LEU 214 | 0.018 +/- 0.010 | THR 98 | -2.513 +/- 0.500 | LEU 214 | -0.072 +/- 0.012 |
| SER 99 | 0.217 +/- 0.084 | HIE 215 | 0.034 +/- 0.035 | SER 99 | -3.125 +/- 0.457 | HIE 215 | -0.118 +/- 0.030 |
| ILE 100 | -0.018 +/- 0.033 | MET 216 | 0.103 +/- 0.011 | ILE 100 | -1.002 +/- 0.489 | MET 216 | -0.100 +/- 0.042 |
| GLU 101 | 0.422 +/- 0.025 | LYS 217 | -0.169 +/- 0.014 | GLU 101 | 0.847 +/- 0.045 | LYS 217 | -0.189 +/- 0.004 |
| ASN 102 | 0.159 +/- 0.021 | GLU 218 | 0.494 +/- 0.015 | ASN 102 | 0.036 +/- 0.012 | GLU 218 | 0.376 +/- 0.010 |
| ARG 103 | -0.252 +/- 0.004 | HIE 219 | 0.033 +/- 0.021 | ARG 103 | -0.298 +/- 0.008 | HIE 219 | 0.062 +/- 0.008 |
| VAL 104 | -0.089 +/- 0.023 | PRO 220 | 0.066 +/- 0.008 | VAL 104 | -0.235 +/- 0.046 | PRO 220 | 0.049 +/- 0.004 |
| ARG 105 | -1.704 +/- 0.357 | ASP 221 | 0.429 +/- 0.006 | ARG 105 | -10.025 +/- 0.831 | ASP 221 | 0.467 +/- 0.006 |
| GLY 106 | 0.022 +/- 0.006 | TYR 222 | 0.154 +/- 0.048 | GLY 106 | 0.019 +/- 0.008 | TYR 222 | -0.033 +/- 0.044 |
| ASN 107 | 0.013 +/- 0.009 | LYS 223 | -1.451 +/- 0.158 | ASN 107 | 0.032 +/- 0.011 | LYS 223 | -7.191 +/- 0.832 |
| LEU 108 | -0.252 +/- 0.070 | TYR 224 | -2.143 +/- 0.605 | LEU 108 | -0.131 +/- 0.016 | TYR 224 | -3.490 +/- 0.573 |
| GLU 109 | 0.739 +/- 0.047 | ARG 225 | -1.556 +/- 0.225 | GLU 109 | 1.620 +/- 0.152 | ARG 225 | -1.669 +/- 0.227 |
| ASN 110 | 0.022 +/- 0.004 | PRO 226 | -7.498 +/- 0.229 | ASN 110 | 0.024 +/- 0.007 | PRO 226 | -7.326 +/- 0.398 |
| LEU 111 | -0.015 +/- 0.013 | ARG 227 | 13.352 +/- 1.317 | LEU 111 | -0.037 +/- 0.013 | ARG 227 | 12.108 +/- 0.852 |
| PHE 112 | -0.035 +/- 0.026 | ARG 228 | -9.532 +/- 0.412 | PHE 112 | -0.092 +/- 0.016 | ARG 228 | 16.337 +/- 1.329 |
| LEU 113 | 0.004 +/- 0.005 | LYS 229 | -6.232 +/- 0.751 | LEU 113 | 0.014 +/- 0.005 | LYS 229 | -5.362 +/- 0.476 |
| GLN 114 | 0.029 +/- 0.004 | THR 230 | 0.243 +/- 0.027 | GLN 114 | 0.056 +/- 0.003 | THR 230 | -0.474 +/- 0.162 |
| CYS 115 | 0.063 +/- 0.011 | LYS 231 | -7.763 +/- 0.471 | CYS 115 | 0.004 +/- 0.015 | LYS 231 | -5.394 +/- 0.914 |
| PRO 116 | -0.028 +/- 0.018 | THR 232 | -1.625 +/- 1.082 | PRO 116 | 0.022 +/- 0.012 | THR 232 | 1.897 +/- 0.119 |
