# Supplementary material for: Structural Mechanism behind Distinct Efficiency of Oct4/Sox2 Proteins in Differentially Spaced DNA Complexes
Source: PLoS One. 2016 Jan 20;11(1):e0147240. doi: 10.1371/journal.pone.0147240 (PMC4720428; doi:10.1371/journal.pone.0147240)
Supplement: S4 Table — (DOCX) [file pone.0147240.s009.docx]

**S4 Table. Binding free energy of Oct4/Sox2^0bp^ and Oct4/Sox2^3bp^ complexes, as determined using g_mmpbsa (GROMACS tool).**

| **Energy Terms (kcal mol^-1^)** | **Oct4/Sox2^0bp^** | **Oct4/Sox2^3bp^** |
| --- | --- | --- |
| van der Waal energy | -327.16 ± 13.029 | -339.0 ± 16.27 |
| Electrostatic energy | -16983 ± 209.5 | -18958.1 ± 220.9 |
| Polar solvation energy | 1164.17 ± 50.28 | 1341.32 ± 55.6 |
| SASA energy | -33.815 ± 1.036 | -36.6 ± 1.08 |
| SAV energy | -394.186 ± 15.41 | -429.3 ± 20.7 |
| WCA energy | 516.69 ± 3.77 | 527.5 ± 4.9 |
| Binding energy | -16057.455 ± 187.86 | -17894.21 ± 206.11 |
